# Whole body regeneration and developmental competition in two botryllid ascidians

**DOI:** 10.1186/s13227-021-00185-y

**Published:** 2021-12-15

**Authors:** Shane Nourizadeh, Susannah Kassmer, Delany Rodriguez, Laurel S. Hiebert, Anthony W. De Tomaso

**Affiliations:** Department of Molecular, Cellular and Developmental Biology, University of California, Santa Barbara, 93106 USA

**Keywords:** Regeneration, Cell competition, Developmental competition, Stem cells, Asexual development, Coloniality, Niche

## Abstract

**Background:**

Botryllid ascidians are a group of marine invertebrate chordates that are colonial and grow by repeated rounds of asexual reproduction to form a colony of individual bodies, called zooids, linked by a common vascular network. Two distinct processes are responsible for zooid regeneration. In the first, called blastogenesis, new zooids arise from a region of multipotent epithelium from a pre-existing zooid. In the second, called whole body regeneration (WBR), mobile cells in the vasculature coalesce and are the source of the new zooid. In some botryllid species, blastogenesis and WBR occur concurrently, while in others, blastogenesis is used exclusively for growth, while WBR only occurs following injury or exiting periods of dormancy. In species such as *Botrylloides diegensis*, injury induced WBR is triggered by the surgical isolation of a small piece of vasculature. However, *Botryllus schlosseri* has unique requirements that must be met for successful injury induced WBR. Our goal was to understand why there would be different requirements between these two species.

**Results:**

While WBR in *B. diegensis* was robust, we found that in *B. schlosseri*, new zooid growth following injury is unlikely due to circulatory cells, but instead a result of ectopic development of tissues leftover from the blastogenic process. These tissues could be whole, damaged, or partially resorbed developing zooids, and we defined the minimal amount of vascular biomass to support ectopic regeneration. We did find a common theme between the two species: a competitive process exists which results in only a single zooid reaching maturity following injury. We utilized this phenomenon and found that competition is reversible and mediated by circulating factors and/or cells.

**Conclusions:**

We propose that WBR does not occur in *B. schlosseri* and that the unique requirements defined in other studies only serve to increase the chances of ectopic development. This is likely a response to injury as we have discovered a vascular-based reversible competitive mechanism which ensures that only a single zooid completes development. This competition has been described in other species, but the unique response of *B. schlosseri* to injury provides a new model to study resource allocation and competition within an individual.

**Supplementary Information:**

The online version contains supplementary material available at 10.1186/s13227-021-00185-y.

## Background

**Fig. 1:**
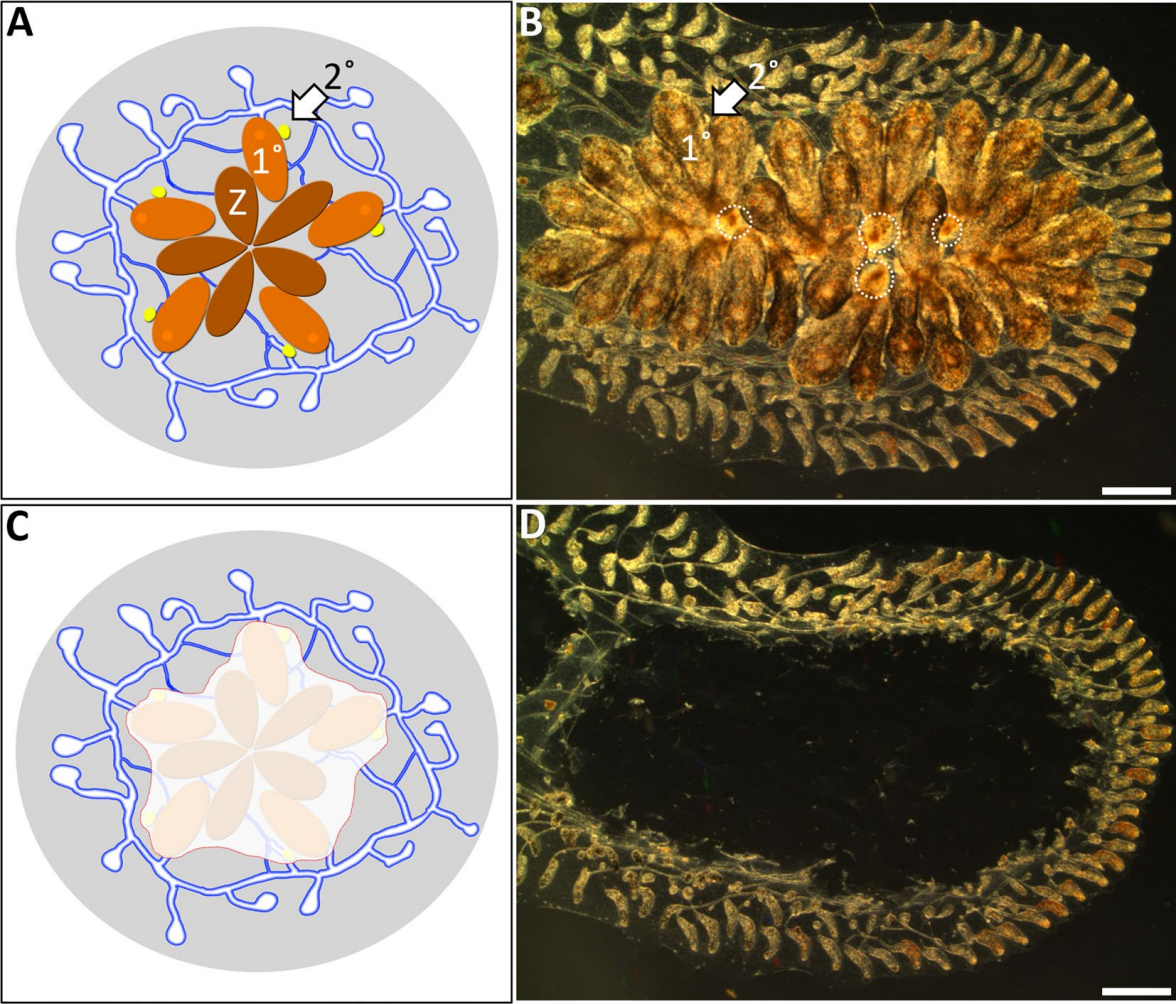
Surgery performed on *Botryllus schlosseri* to induce whole body regeneration. **A** Illustration showing a system of *B. schlosseri*. Depicted are zooids in brown (Z), primary buds in orange (1°), and secondary buds in yellow (2°). **B** Pre-surgery darkfield image of a colony growing on a glass slide. Zooids are almost entirely resorbed (shown in white dashed circles). **C** Illustration shows excision of zooids and primary/secondary buds, but vascular tissue remains intact. **D** Post-surgery image of same colony in **B** 2 min following the removal of all zooids and developing bud tissues. Scale bars = 1.0 mm

Ascidians (subphylum Tunicata) are marine chordates and the closest living invertebrate relatives to vertebrates [1]. Sexual reproduction leads to a pelagic chordate tadpole larva that swims to find a suitable substrate [2], then settles and undergoes a metamorphosis to a sessile adult individual. The resulting invertebrate body plan is called an oozooid and has little resemblance to the larval form [2–4]. The oozooid is a filter feeder with a complex body plan that includes incurrent and excurrent siphons, a central and peripheral nervous system, a pharynx for respiration and feeding, stomach, intestine, gonads, circulatory system, tube-like heart, and glands for endocrine signaling [5–8]. After metamorphosis, there are two divergent life histories among the ascidian species. Some species, such as *Ciona robusta*, grow by increasing in size and become sexually mature during their 1-year lifespan. However, many species are colonial [9] and propagate asexually. This process, called budding, generates multiple independent individuals (called zooids) with a similar body plan to that of the oozooid [10–14]. Budding occurs throughout the life of a colony and can lead to thousands of clonal zooids, all derived from a single larva [15]. In the botryllids (*Botryllus* and *Botrylloides* genera), all adult and developing zooids (buds) are connected by a common vasculature that ramifies throughout the colony. Outside of the bodies, vessels radiate distally and terminate into close-ended oval shaped structures called ampullae that can be found throughout the tunic and concentrated at the colony periphery (Fig. 1A and B). All bodies and blood vessels are embedded within a cellulose-based tunic, a defining feature of this phylum. Among the colonial species there are multiple asexual budding pathways that have arisen independently, and the diversity and phylogeny of these processes have been extensively reviewed [16–18].

Individual genotypes of colonial ascidians such as *Botryllus schlosseri* and *Botrylloides diegensis* can consist of thousands of zooids and survive for years; however, the zooids (Additional file 1: Fig. S1A, labeled Z1–3) are short-lived. In these species, new zooids regenerate weekly through a process called blastogenesis (Additional file 2: Video S1). Blastogenesis in most botryllid species is organized into three concurrent generations that are spatially arranged and developing synchronously (Additional file 1: Fig. S1B). Zooids, the oldest generation, are the only individuals in a colony with an open oral and atrial siphon for filter feeding and sexual reproduction. Primary buds are the second oldest generation and are found proximal to the zooids; they are in the last stages of development (Additional file 1: Fig. S1C–F, white arrow). Secondary buds are the youngest generation and located proximal to the primary bud (Additional file 1: Fig. S1D–F, black arrow). Secondary buds originate from a region of the primary bud called the peribranchial epithelium, and buds originally derived from this tissue are called peribranchial buds. At the end of each blastogenetic cycle, all adult zooids die and are resorbed through a coordinated process of apoptosis and phagocytosis known as takeover [19, 20]. Primary buds migrate to the newly vacated area, open their siphons, and begin feeding, defining them as adult zooids. Secondary buds become primary buds and continue development, and new secondary buds initiate development (Additional file 2: Video S1). In *B. schlosseri*, zooids are arranged into star-shaped groups known as systems, with zooids occupying the center, and primary buds and secondary buds being distal, respectively. In *B. diegensis*, the budding process is the same, but the zooids are arranged linearly.

In colonial species of the family Styelidae, in which *B. schlosseri* and *B. diegensis* are classified, another asexual budding mode exists, called vascular budding. Here the source of the new bud is a population of circulatory cells, which aggregate within the blood vessels, form into a blastula-like structure, then develop into a new bud in situ in a process morphologically equivalent to peribranchial budding, even down to the number of rows of stigmata [13]. Both peribranchial and vascular budding are utilized differently among botryllid species. For example, *Botryllus primigenus* and *Botryllus tuberatus,* can simultaneously form peribranchial and vascular buds [11, 13, 21, 22]. Others, like *Botrylloides violaceus*, *B. diegensis*, and *B. schlosseri*, undergo colony expansion exclusively through peribranchial budding [11].

**Fig. 2:**
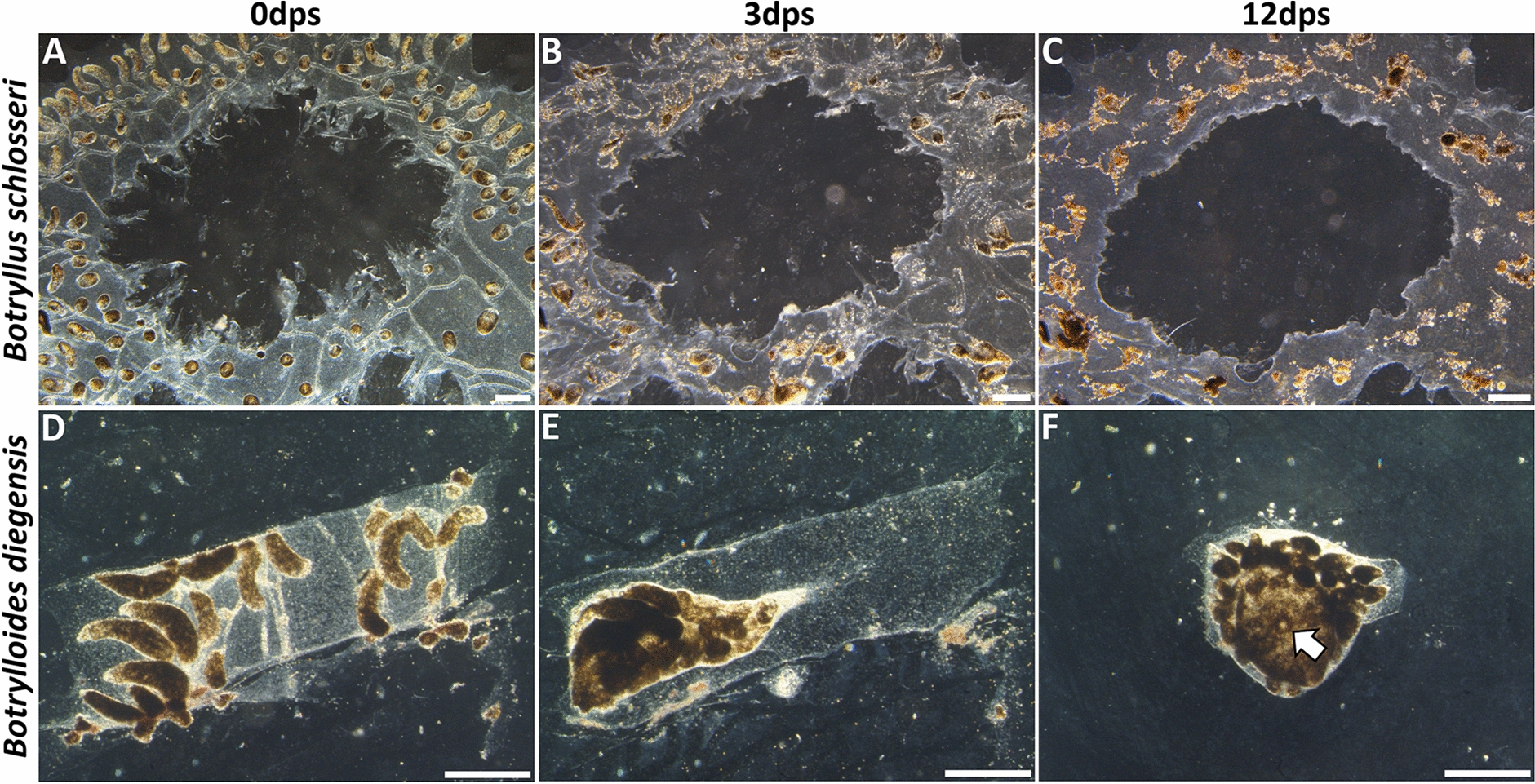
Comparison of whole body regeneration (WBR) surgeries between *Botryllus schlosseri* and *Botrylloides diegensis*. **A** Darkfield image of *B. schlosseri* after surgery. Ten zooids were excised, and blood circulation remained strong throughout vasculature. **B** After 3 days, blood continued to flow, and vasculature had rearranged within the tunic. **C** After 12 days, blood flow had stopped, and the vasculature showed increased pigmentation. No sign of WBR, and vessel migration had ceased. **D** Darkfield image of *Botrylloides diegensis* after surgery. Minimal vasculature and approximately 20 ampullae were left after removal of zooids and buds. **E** Three days post-surgery the vasculature had condensed. **F** At day 12 post-surgery an open oral siphon (arrow) and atrial siphon have developed. Ampullae have begun to spread outward and sexual budding resumes. *dps* days post-surgery. Scale bars 0.5 mm

Vascular budding is also observed in two other situations in the botryllids: response to injury and exit from seasonal dormancy. When vascular budding is induced by injury, it is also referred to as whole body regeneration (WBR), and only occurs following a surgical stimulus that involves isolating portions of the extracorporeal blood vasculature away from, or ablating, all zooids and buds [11, 23–30] (Fig. 2). This stimulus triggers vascular rearrangement and initiates blood cell aggregation, the first step of vascular budding. The zooid that develops then switches back to peribranchial budding, eventually regenerating the entire colony. Vascular budding is also utilized to exit seasonal dormancy. Environmental perturbations trigger dormancy, which cause zooids and developing peribranchial buds to resorb and blood vessels to coalesce until conditions improve [31]. Dormancy can last for months, and during that time the colony resembles the early stages of WBR; the vessels have remodeled into an opaque mat and multiple presumptive vascular buds at the earliest stages of development—aggregates and blastula-like structures—are present [31, 32]. When environmental conditions return to normal, these presumptive buds complete development, begin feeding, and initiate peribranchial budding to regenerate the colony.

We have been coupling transplantation and prospective isolation studies to identify the cells which initiate WBR [24], and one of our aims was to compare this process between *B. schlosseri* and *B. diegensis.* One interesting observation is that the requirements for WBR in the two species are very different. In *B. diegensis*, WBR is easy and robust; simply isolating a small 2 mm^2^ area of peripheral vasculature in any stage will trigger vascular bud development, and a zooid will develop to maturity in around 10 days with an efficiency > 90% [24]. In contrast, previous publications in *B. schlosseri* from multiple labs around the world have found that WBR requires several strict conditions. These include using an entire vascular network from a large colony—a size that is ten times larger than that required for *B. diegensis*—and that the surgical removal of the zooids and buds must occur during a specific 36 h window during takeover (Additional file 2: Video S1, 155–165 h). If these requirements are not met, WBR is not successful [14, 25, 33, 34].

Previous studies have clearly shown that WBR in *Botrylloides* occurs from the vasculature [26, 28, 30], and that the source for development is a population of circulating cells [24]. In contrast, none of the previous studies in *B. schlosseri* show clear longitudinal evidence of a bud developing within the transparent vasculature.

The initial goal of this study was to rigorously analyze the early stages of WBR in *B. schlosseri* to determine the source of the new bud and try to understand why the requirements for successful regeneration were much more stringent*.* In our experiments, we found that the zooid which develops after surgical isolation of blood vessels in *B. schlosseri* is not due to circulatory cells initiating WBR, but instead relies upon ectopic development of remnants of the blastogenic process. While analyzing the timing and activity of ectopic development in *B. schlosseri* we also found that if multiple buds survived surgery, that they would compete for survival with only one winning and completing development. An analogous competition between developing vascular buds has also been shown to occur during WBR in *B. leachii* [28]. We followed up on these observations and discovered that a reversible mechanism of competition exists in *B. schlosseri* and is mediated through the vascular network.

## Results

### Disparities in injury response between phylogenetically related species

Previous studies on whole body regeneration (WBR) in *Botryllus schlosseri* concluded that there were three requirements for zooid development from isolated vasculature: (1) experimental colonies must be large, having nine or more zooids [34]; (2) the marginal vessel (central blood vessel that connects all zooids and ampullae; Additional file 3: Fig. S2), must be left intact following ablation of the zooids and buds for colony-wide circulation (Additional file 4: Video S2) [14]; and, (3) surgery required ablation of the zooids and buds when the zooids are resorbing during the takeover process [33, 34]. To make sense of the disparate requirements between this species and *B. diegensis*, we attempted to replicate previous experiments in *B. schlosseri* by carefully removing zooids and developing buds from large colonies to isolate blood vasculature and induce WBR (Fig. 1). We made detailed observations by carrying out longitudinal studies and recording timelapse videos starting immediately following surgery. While collecting data for both species, *B. schlosseri* and *B. diegensis* (Fig. 2), we never observed a zooid developing from an isolated blood vessel in *B. schlosseri* (Fig. 2C). In contrast, zooid development in *B. diegensis* was robust (Fig. 2F).

In both species, the vascular network initially reacted to colony damage by rapidly clotting up severed vessels to prevent blood loss. Next, the vasculature actively remodeled within the tunic matrix, with major differences observed between the two species. After 3 days of reorganization, the tissues in *B. diegensis* coalesced into a compact mass (Fig. 2E). In contrast, *B. schlosseri* vessels went through a characteristic global regression, followed by vessel re-extension toward the colony periphery (Additional file 5: Video S3). This retraction and re-extension process is consistent among genotypes and takes approximately 24 h.

### Unremoved secondary buds migrate to vasculature and continue development

After twelve timelapse experiments with *B. schlosseri* we observed a WBR event following zooid ablation (Additional file 6: Video S4). However, through retrospective analyses of high-resolution images, we noticed small developing bud tissues had been inadvertently left behind following surgery. The observed tissues migrated away from their original location through the tunic, and restored contact with the peripheral blood vessel. Once fused with the circulatory system, these tissues increased in size and continued to develop as if seemingly derived from the blood vasculature.

To follow up on these results, we performed over 150 surgeries to ensure removal of all zooids and developing bodies from large, stage D colonies of *B. schlosseri* (Additional file 7: Fig. S3A–C). Experiments included five distinct genotypes from the Santa Barbara harbor on the Pacific coast of California (Additional file 8: Table S1). We only scored animals that restored colony-wide circulation and showed robust blood flow throughout the observation window (*n* = 128); therefore, in over 85% of our experiments we analyzed vascular rearrangement and blood circulation for up to 12 days following surgeries. None of these experiments provided evidence that WBR could be induced through injury. Instead, we observed characteristic vascular remodeling (described above), followed by eventual constriction of vessels, cessation of blood flow, and necrosis of remaining tissues (Additional file 7: Fig. S3D–F). If a zooid developed from the vasculature, we could visually identify its origins outside of the vasculature using stereoscope micrographs. We also carried out whole mount in situ hybridization of the vasculature following surgery to see if cell aggregates were forming. In these experiments, we used a probe for the pluripotency marker *pou3* [24]*,* and counterstained with an antibody to phosphohistone H3 (a mitotic marker) and the nuclear stain DAPI, which together would allow us to see aggregations of any cell type. While these markers clearly identified both aggregations and blastula-like structures consisting of proliferating *pou3*+ cells in *B. diegensis* [24], we never identified any clusters of *pou3*+ or proliferating cells in *B. schlosseri*, at any time point (Additional file 9: Fig. S4). Even when we performed surgeries on very large colonies, > 4× the reported minimal size requirement (*n* = 8), there was no indication of regeneration (Additional file 10: Fig. S5). In contrast, *B. diegensis* robustly and repeatedly underwent WBR from minimal vascular tissue (Fig. 2D–F).

**Fig. 3:**
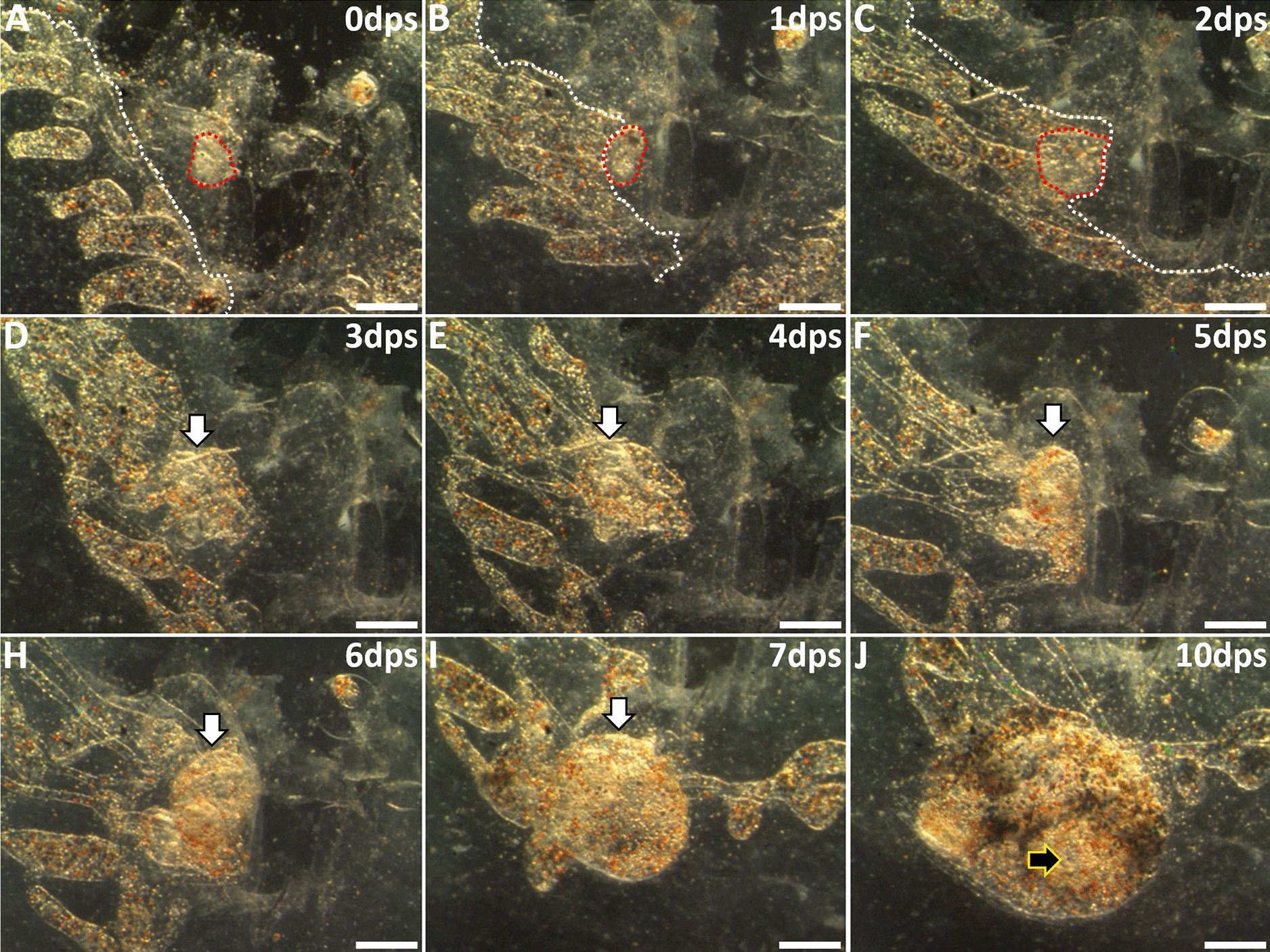
Secondary bud migration and development following removal of zooids and other developing buds. **A** Post-surgery darkfield image showing vasculature (demarcated by white dashed line), tunic, and a single remaining stage D secondary bud (outlined in red dashed line). **B** After 24 h, the secondary bud is adjacent to the vasculature. **C**, **D** At 2- and 3-days post-surgery, the secondary bud continued development and had merged with the vasculature. Subsequent panels indicate location of secondary bud (white arrows). **E**–**I** The secondary bud shows pigmentation and is now evident under the microscope. The heartbeat developed during this 5–7-day time frame which allowed for easy detection. **J** Ten days post-surgery an open siphon is visible (black arrow) and the zooid was actively feeding. *dps* days post-surgery. Scale bars = 250 µm

In summary, when zooids developed after surgery in *B. schlosseri*, we could always retrospectively identify a previously undetected transparent tissue that was outside of the vasculature following surgery, but rapidly migrated and re-attached to the vasculature as the source of the new zooid (Table 1, Fig. 3, Additional file 6: Video S4). This tissue initially appeared near the peripheral vasculature and were most likely secondary buds that we missed during surgical ablation. At this point in the blastogenic cycle, secondary buds are small (250 × 100 µm), and lack pigmentation. It would be easy to miss ablating them, particularly since the peripheral vasculature cannot be damaged for WBR to occur; thus, one would avoid cutting close to the vessel (Fig. 2A). We found the most critical time point of these observations were the initial hours after surgery, during which we observed tissues migrating from their original position to fuse with vasculature (Fig. 3A–C). This phenotypically appeared as though a zooid developed directly from the remaining vasculature (Fig. 3D–J), but we could always predict where the zooid would arise following surgery when detailed images were scrutinized for migrating tissues.

**Table 1:** The potential of various body tissues (whole or partial) to develop into a feeding zooid

Tissues tested with vasculature	Open siphon (%)	*N*
None (Fig. 1C, D)	0	128
Partial primary bud (data not shown)	0	20
Secondary + partial primary bud (Fig. 4A, B)	89	82
Secondary bud (Fig. 4D, E)	48	27
Fragmented secondary bud (Additional file 14: Fig. S8A, B)	6	16
Secondary bud with reduced vasculature (≤ 3 mm^2^) (Fig. 5D)	0	10
Secondary bud with reduced vasculature (~ 6 mm^2^) (Fig. 5H)	25	4

Seven surgery permutations were performed on *Botryllus schlosseri* to determine how much tissue was required for a zooid to develop after injury, as assessed by the opening of siphons and feeding. The figures mentioned in column 1 are representative images for each surgery. Vascular tissue and tunic alone were insufficient to recover from loss of all zooids and associated buds. When only the anterior region of a primary bud was left to develop, it was resorbed into the existing tissue, but no zooid formation occurred. When a secondary bud was associated with that same portion of the primary bud, a zooid developed. Furthermore, secondary buds alone can complete development 48% of the time, but if damaged, survivability drops to only 6%. Secondary buds did not survive with less than 3 mm^2^ tunic area after surgery; however, leaving an area of 6 mm^2^ was sufficient to support full secondary bud development and asexual budding

### Injury and characterizing development of remaining tissues

Developing buds can be near the marginal vessel or situated partially underneath the zooid; thus, it is possible to leave fragments of primary buds with secondary buds after surgery. We followed up on previous observations by removing all zooids at stage D and purposefully leaving combinations of primary and secondary bud tissues to characterize the response. We initially carried out two experiments, leaving only intact secondary buds or leaving fragments of the primary bud coupled to the secondary bud. In both cases, the remaining tissue migrated from its original location, re-attached to the peripheral vasculature, and then completed development into a zooid exactly as we had seen previously. When part of the primary bud was left, it was resorbed by the developing bud, and the zooid developed in 89% of the cases (Additional file 11: Fig. S6). When only a secondary bud was isolated without any anterior primary bud tissue, this decreased survival to open siphon down to 48% (Table 1). Finally, when only a secondary bud was left, in some cases we observed that the resulting zooid had an abnormal phenotype, including being shifted sideways in the tunic, such that the siphons pointed to the left or right, rather than dorsally (Additional file 12: Fig. S7) [34].

**Fig. 4:**
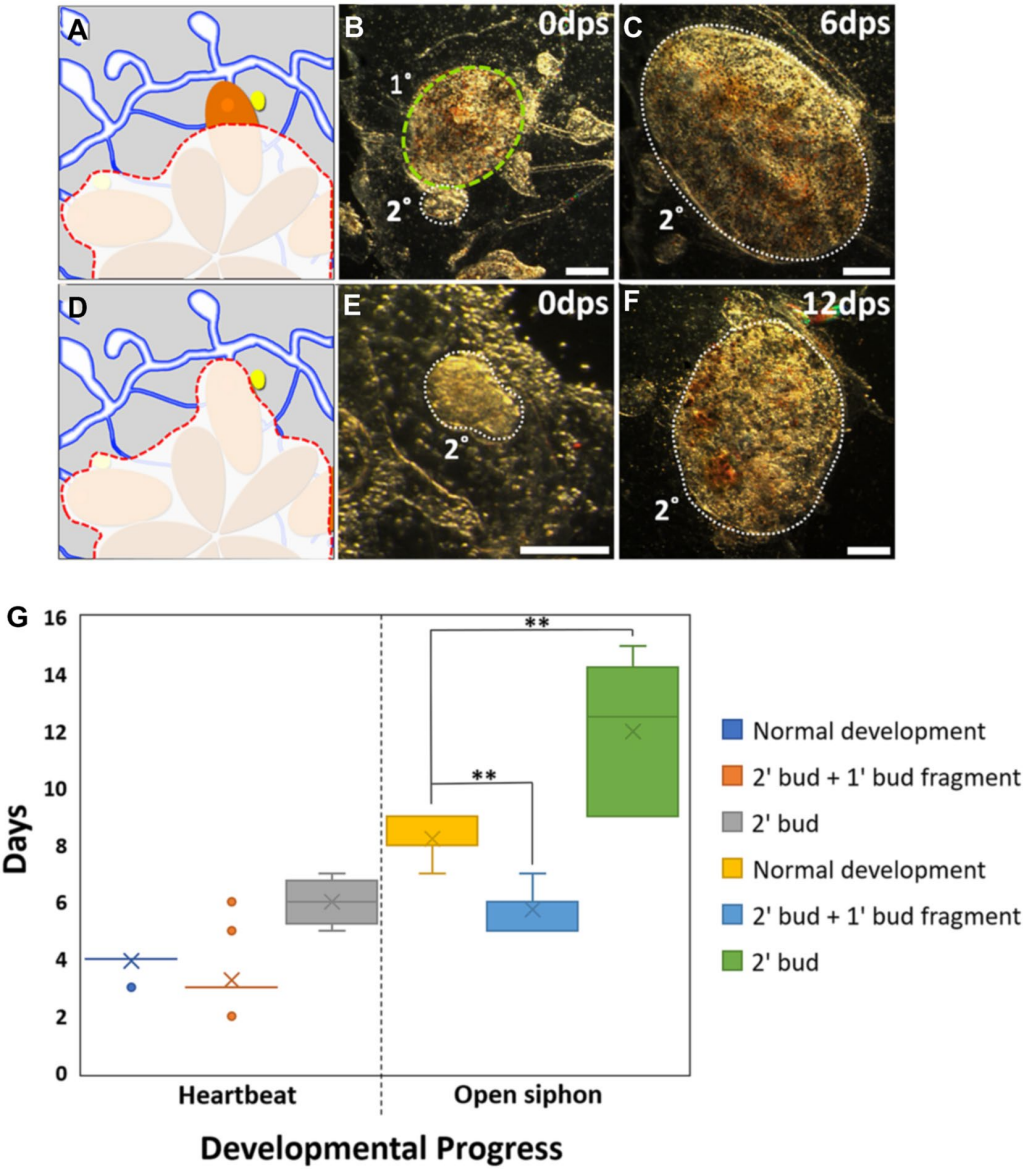
Post-surgery timing of secondary bud development. **A** Illustration showing surgery to isolate secondary bud (yellow) and anterior region of associated primary bud (orange). Excised tissues in red dashed outline. **B**, **C** Post-surgery darkfield images of those bud tissues at day 0 and 6, respectively. Secondary bud is outlined with white dotted line. Portion of primary bud is outlined with yellow dashed line. **D** Illustration showing surgery performed to isolate a secondary bud alone (yellow). **E**, **F** Post-surgery darkfield images of secondary bud tissue at day 0 and 12, respectively. The siphon opened at day 13. **G** Quantitative results comparing the timing of development to heartbeat and siphon opening during normal budding, secondary/primary bud, and secondary bud alone. *dps* days post-surgery. Asterisks indicate statistical significance (**P < 0.01). Scale bars = 0.25 mm

One interesting observation regarded differences in the timing of development following these two surgeries. During normal peribranchial budding in lab-reared colonies, stage D secondary buds which are 6 days old will form a pumping heart 3 days later (day 9) and the siphon will open 5 days after the heartbeat initiates (day 14). When we observed development following surgeries in which the secondary bud remained attached to the primary bud anterior region (Fig. 4A), it required on average 3 days for heart formation and 6 days to open a siphon (Fig. 4C). Thus, secondary buds developed at a normal pace when remaining primary bud tissues were present. When we performed surgeries to leave only the secondary bud (Fig. 4D, E), it required 6 days for hearts to pump, and 12 days for siphon opening (Fig. 4F), an approximately twofold delay vs unmanipulated peribranchial budding (Fig. 4G, Additional file 13: Table S2). These are similar ranges described for heart beat initiation and siphon opening to occur in previous WBR studies [14, 25, 33, 34].

If WBR in *B. schlosseri* is due to tissues leftover by accident, it would not be in the controlled fashion utilized in the previous experiments. We next characterized the level of damage that could occur to a secondary bud and still result in zooid development. We removed all but one intact secondary bud from a large colony at stage D, then injured that bud and observed the results. Our experimental injury applied pressure to the tunic above the bud without tearing into the tunic, until gross morphology was perturbed (Additional file 14: Fig. S8A, B). While the cells do aggregate prior to migration, they do not form the same tight association as that of an undamaged secondary bud (Fig. 4D, E). The reason for this method was because secondary buds did not survive direct surgical cuts. Interestingly, 25% of damaged secondary buds developed a pumping heart, but only in 1/16 cases did we observe the damaged bud develop into a mature zooid with open siphons. While this injury model is not replicating what may have happened in previous studies, it does suggest that a relatively undamaged secondary bud is required to generate a zooid. These experiments also provide strong evidence that WBR does not occur in *B. schlosseri*: a single secondary bud was purposefully left and damaged prior to revascularization, but no WBR event was observed under these controlled conditions. Importantly, in the case where development did occur, it did so from the damaged bud.

### Isolated secondary bud survival has a vascular tissue size requirement

Previous studies reported that a continuous marginal blood vessel and approximately 10× more vascular area was required for WBR in *B. schlosseri* versus *B. diegensis.* Additionally, the ablation must take place during takeover, when adult zooids are dying and being phagocytosed in stage D (Additional file 2: Video S1, 155–166 h). Taken together, this suggested that *B. schlosseri* required more energy via catabolism of the remaining tissue versus *B. diegensis*, where only a small portion of the vasculature is required to support WBR (Fig. 2).

**Fig. 5:**
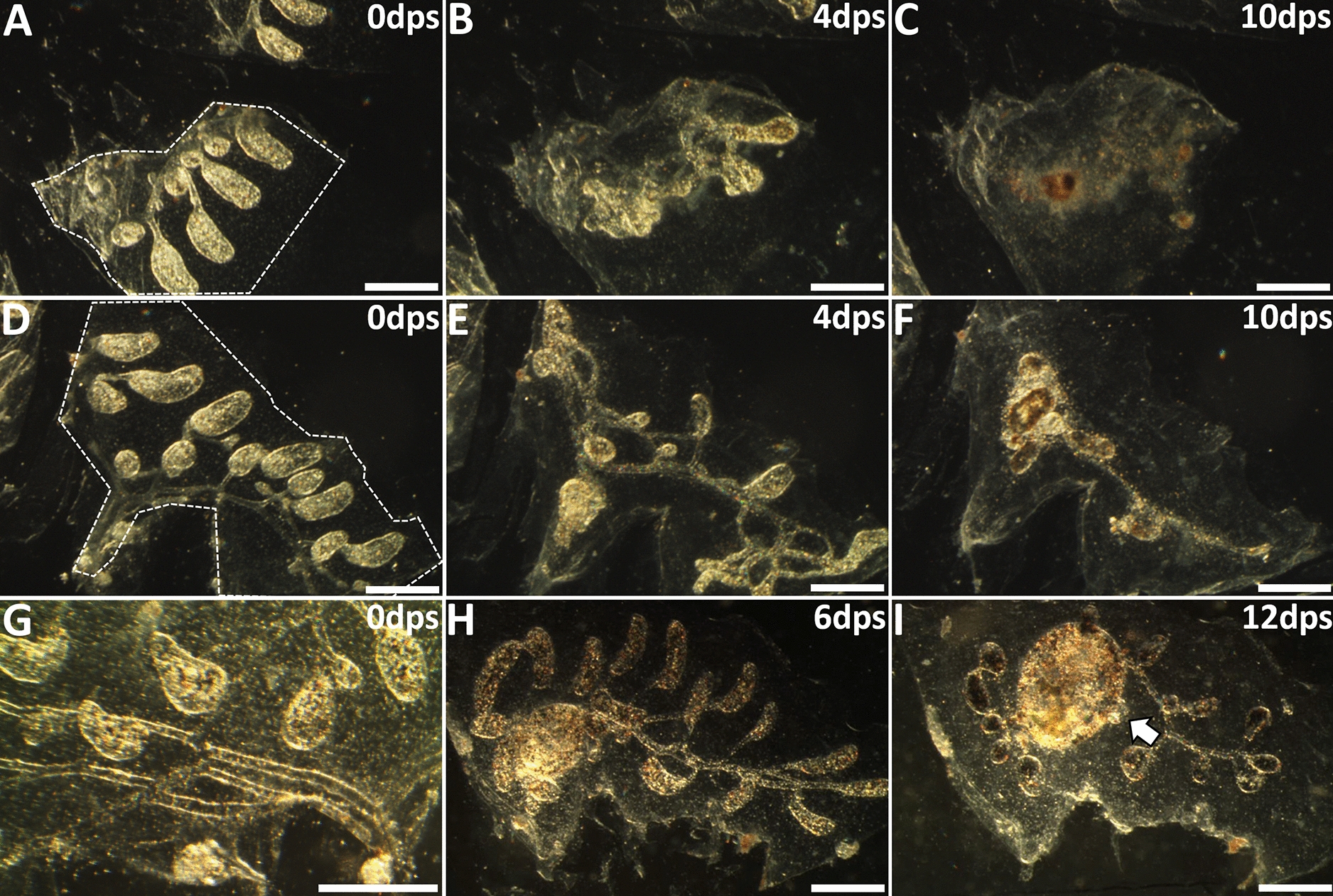
Complete post-surgery secondary bud development has a vasculature size requirement. **A**–**C** Secondary buds that were left to develop with tunic area of 1.5 mm^2^ did not survive and loss of all activity (and necrosis of tissue) was seen by day 10. **D**–**F** Vasculature and tunic with approximate area of 3 mm^2^ with a single secondary bud did not survive but showed longer activity than experiments having less tissue resources. **G**–**I** Full development of a secondary bud was observed with tunic area of 6 mm^2^ or more. *dps* days post-surgery. Scale bars = 0.5 mm

To address this potential difference in energetic demand, we determined the minimal size of remaining vasculature that was required to facilitate successful post-surgery zooid development of a single secondary bud (Table 1). Secondary buds left with ≤ 3.4 mm^2^ of total tissue area did not survive (Fig. 5A–F), whereas isolated secondary buds (Fig. 5G) with tissue area ≥ 6 mm^2^ (Fig. 5H) developed and continued asexual budding (Fig. 5I; Additional file 15: Video S5). Surprisingly, the time to complete development was equivalent whether we used an entire vascular network (Fig. 1), or only a 6 mm^2^ section (Fig. 5): in other words, an area of vasculature larger than the minimum size did not expedite the developmental process. These data show that there is a vascular tissue size requirement for secondary bud survival, but it is < 10% of the size required for successful WBR as described previously [34].

### Secondary buds compete for sole-survivor

Previous studies on WBR in *B. leachii* have revealed that while multiple vascular buds are initiated following surgery, only a single zooid completes development, and this observation was consistent over a large range of vascular tissue [26, 28]. A single zooid can develop from a 3 mm^2^ fragment (Fig. 2D), so larger fragments around 40 mm^2^ (Additional file 16: Fig. S9) could theoretically support the development of multiple zooids, but that is not observed. This suggests that buds compete for resources during WBR, and previous studies suggested competition occurs at the blastula-like stage [28].

**Fig. 6:**
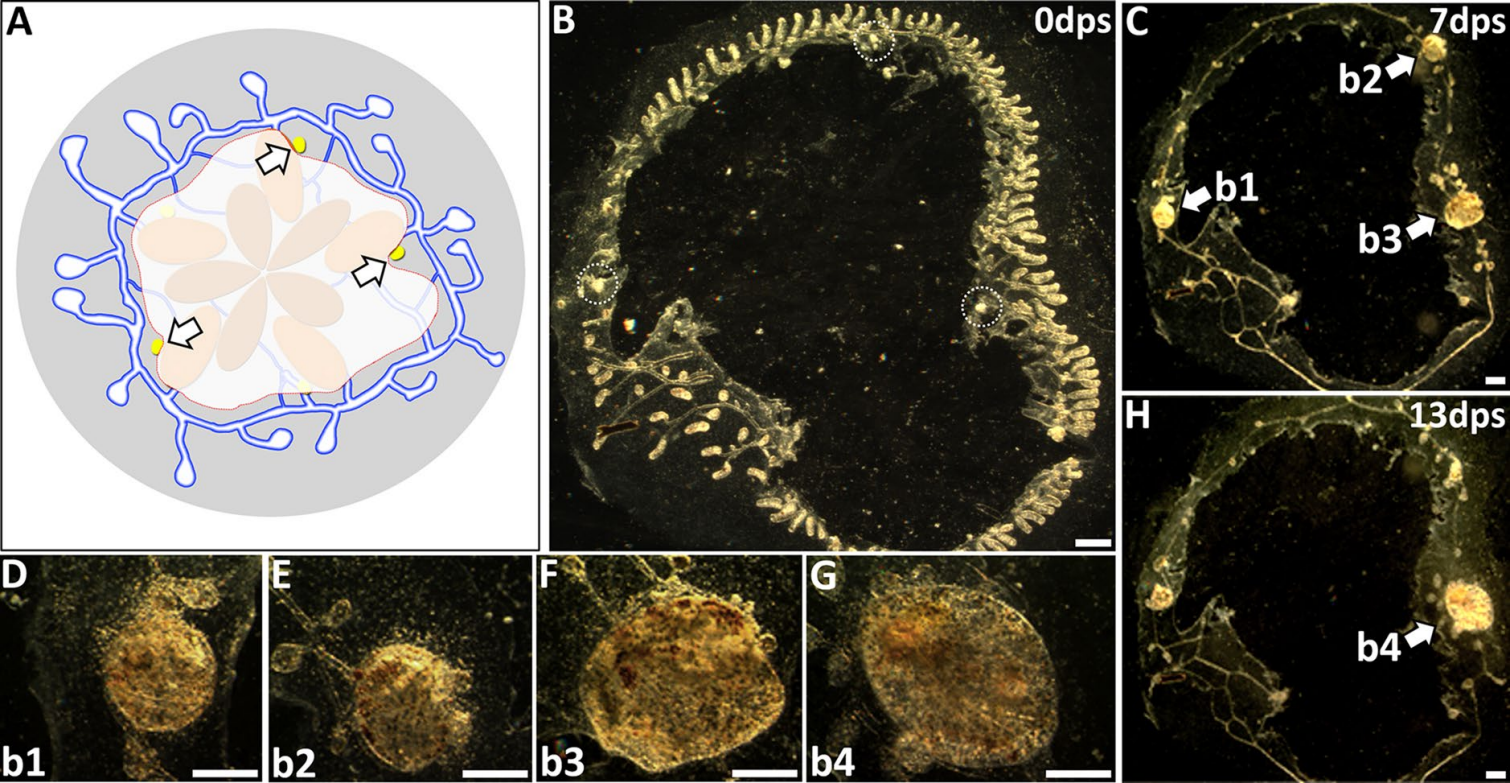
Three post-surgery secondary buds lead to a single zooid through competition in *Botryllus schlosseri*. **A** Illustration showing surgery performed to remove zooids, primary buds, and all but three secondary buds (yellow, white arrows). **B** Darkfield image of post-surgery*.* Secondary buds are indicated (white dashed circles). **C** Seven days post-surgery. All three secondary buds (white arrows) have pumping hearts. Higher magnification of each bud is shown in **D**–**F** along with associated bud # (b1, b2, b3). **D** Bud from left side of **C** (b1). **E** Bud from top of **C** (b2). **F** Bud from right side of **C** (b3). **G** The single zooid that fully developed is shown magnified in **G** (b4, formerly b3). **H** Colony at day 13 showing single zooid (b4, white arrow) had open siphons. Two other buds were in the process of resorption. *dps* days post-surgery. Scale bars = 0.5 mm

**Fig. 7:**
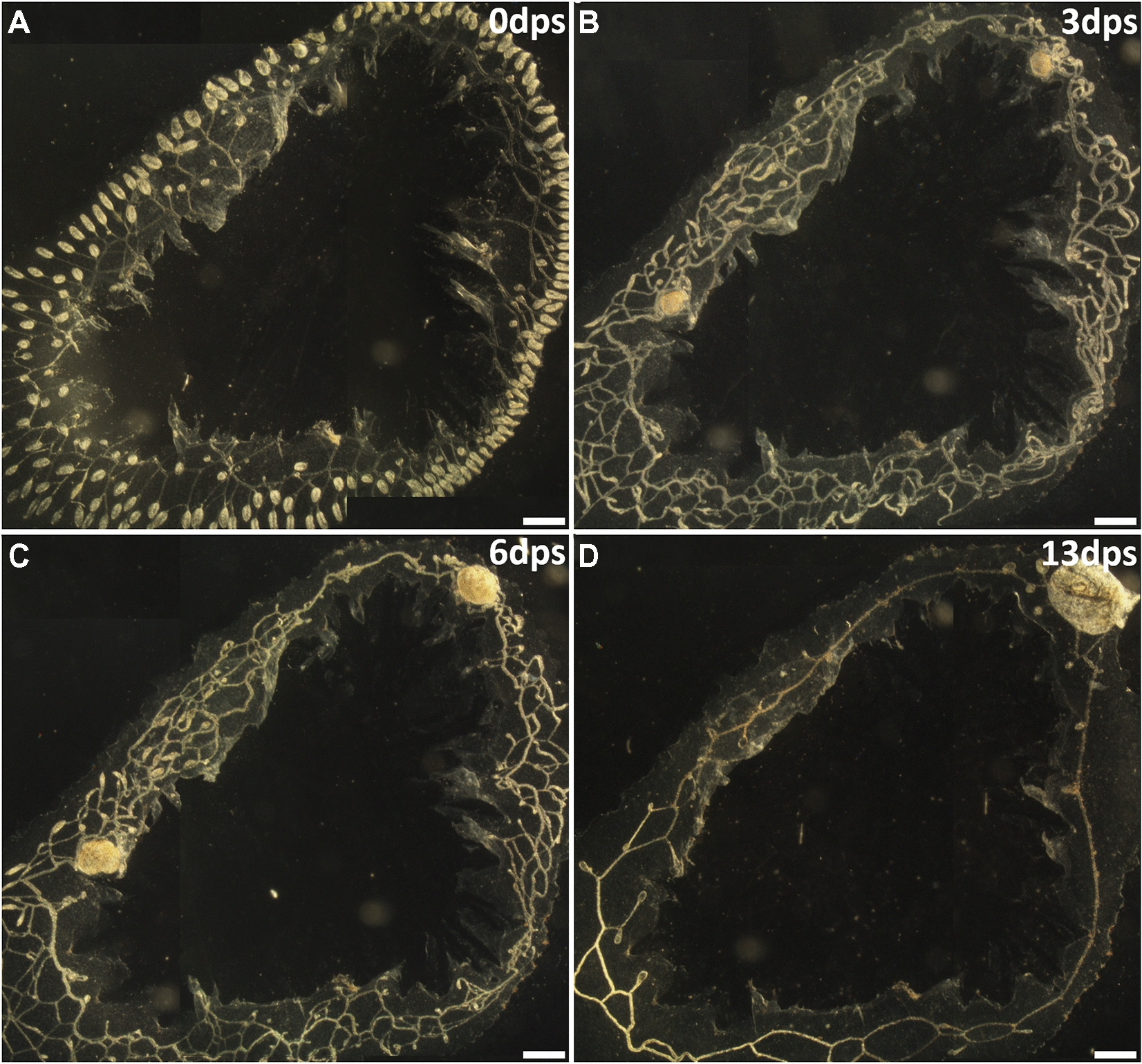
Secondary buds from separate systems can compete. **A** Zooids, primary buds, and all but two secondary buds were removed from a three-system colony. **B** By 3 days post-surgery, both secondary buds had migrated to fuse with the vasculature and commensurately increased in size. **C** On day 6 after surgery, one secondary bud (left side) had reached its maximum size before being developmentally suppressed by the winner secondary bud (right side). **D** A single secondary bud persisted, and the loser secondary bud had completely resorbed. *dps* days post-surgery. Scale bars = 1 mm

If previous results documenting WBR in *B. schlosseri* were actually due to ectopic development of remaining secondary buds, we wondered why these experiments also resulted in the development of only a single zooid [14, 25, 33, 34]. We next asked if competition exists between developing secondary buds in *B. schlosseri*. To assess the presence of interbud competition, we left two or three secondary buds after surgically removing all zooids and primary buds and observed the outcome. When two isolated secondary buds in *B. schlosseri* were left after surgery, the result was a single surviving zooid (*N* = 9). We next examined the outcome when three secondary buds were left (*N* = 3) (Fig. 6). Seven days after surgery, hearts pumped in all three buds, but their sizes varied (Fig. 6C–F). By day 13, only one persisted and opened its siphons to become an active filter-feeding zooid (Fig. 6G). The other two developing secondary buds, which were always sharing blood with the winner, ended up dying and resorbing into the vasculature (Fig. 6H). Because these experiments were done on buds derived from a single system, we next checked if secondary buds originating from separate systems within the same colony could compete, as these larger distances would be more representative of other WBR studies (Fig. 7A). The surgery performed in these experiments left behind two developing buds spaced approximately 1 cm apart (*N* = 3) (Fig. 7B). Both buds developed pumping hearts by day 6 (Fig. 7C) but on day 13 only a single zooid developed, while the other was resorbed (Fig. 7D). Whatever is mediating the interactions between the buds can operate at this distance.

### Competition provides winner secondary bud with more resources

**Fig. 8:**
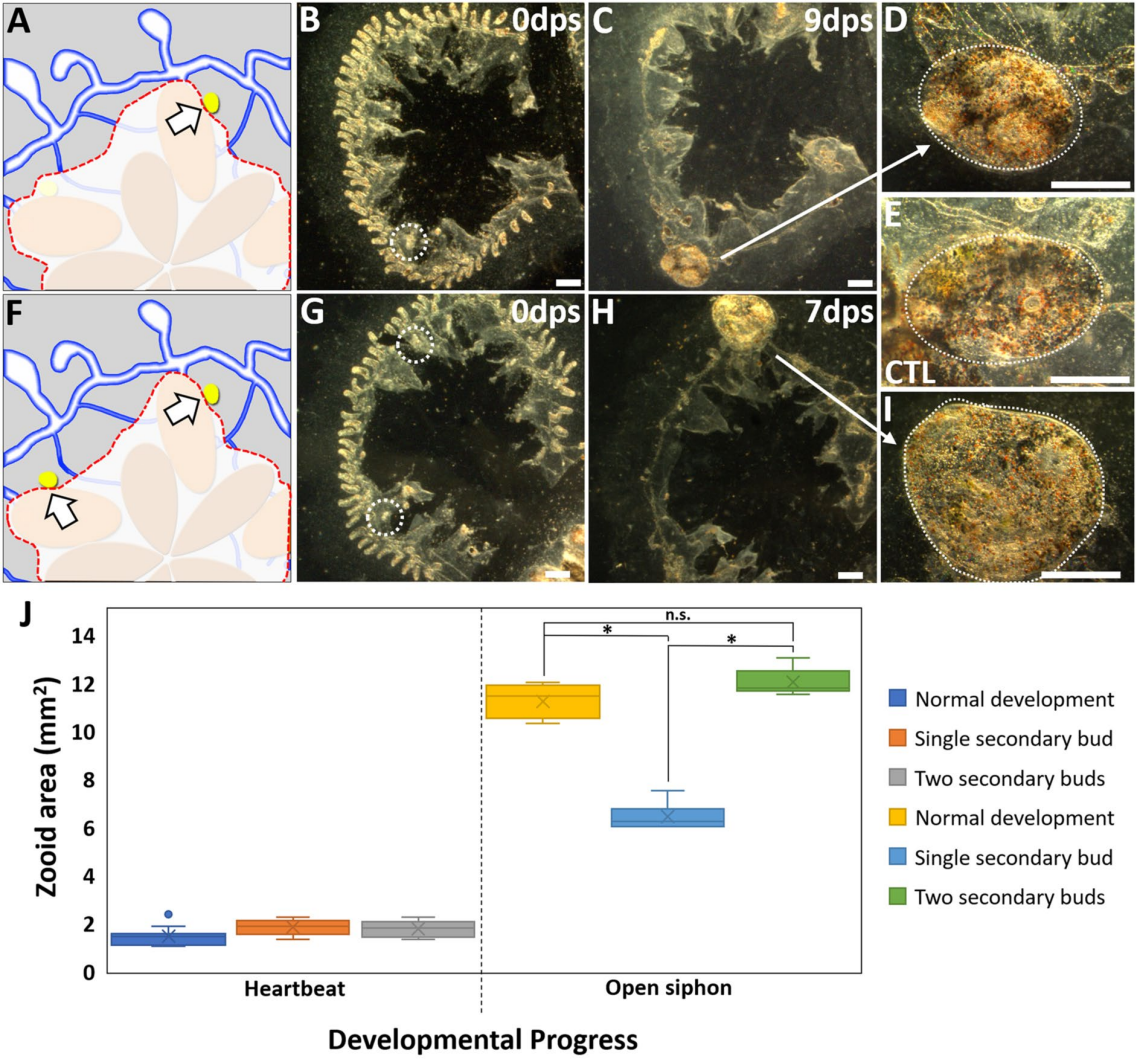
Loser bud resorption increased size of winner bud. **A** Illustration showing surgery performed to isolate secondary bud (white arrow). **B**, **C** Post-surgery darkfield images of secondary bud (white dashed circle) at day 0 and 9, respectively. **D** Higher magnification of zooid in **C** at time of siphon opening. **E** Size of a control zooid upon siphon opening. **F** Illustration showing surgery performed to isolate two secondary buds (white arrows). **G**, **H** Post-surgery darkfield images showing secondary buds (white circles) at day 0 and 7, respectively. **I** Higher magnification of **C** zooid at time of siphon development. **J** Quantitative analysis comparing areas of isolated secondary bud(s) vs. normal/blastogenic bud. Heartbeat and siphon opening used as developmental landmarks. *dps* days post-surgery. Asterisks indicate statistical significance (*P < 0.05). n.s. = not significant. Scale bars = 0.5 mm

A zooid that develops from a single secondary bud (Fig. 8A, B) is significantly smaller (Fig. 8C, D) than a control zooid derived from peribranchial budding (Fig. 8E). However, leaving behind two secondary buds (Fig. 8F, G) gives rise to a single zooid (Fig. 8H, I) that is quantitatively similar in size to the control (Fig. 8J, Additional file 17: Table S3). Interestingly, when multiple secondary buds are left, they commensurately increase in size until the heart begins beating (Fig. 8J). At this point, the non-competitive buds stop growing, begin regressing, and are eventually resorbed. This demonstrates that competition is causing the resorption of loser buds, reallocating those resources to the winner, and that competition is not visually apparent until after the heart has completed development.

### Growth inhibition is due to circulatory factors and is reversible

To narrow down the tissues mediating competition, we did the same experiments leaving two secondary buds, but this time severed the blood vessels 48 h later, when the two buds were approximately equal in size, but prior to the appearance of a functional heart. The shared tunic was left partially intact so that we only disrupted the vascular connections (Additional file 18: Fig. S10B). In this case, secondary buds sharing tunic but not blood both developed to zooids (Additional file 18: Fig. S10D). These findings show that factors in the blood are responsible for the signals driving competition between secondary buds.

**Fig. 9:**
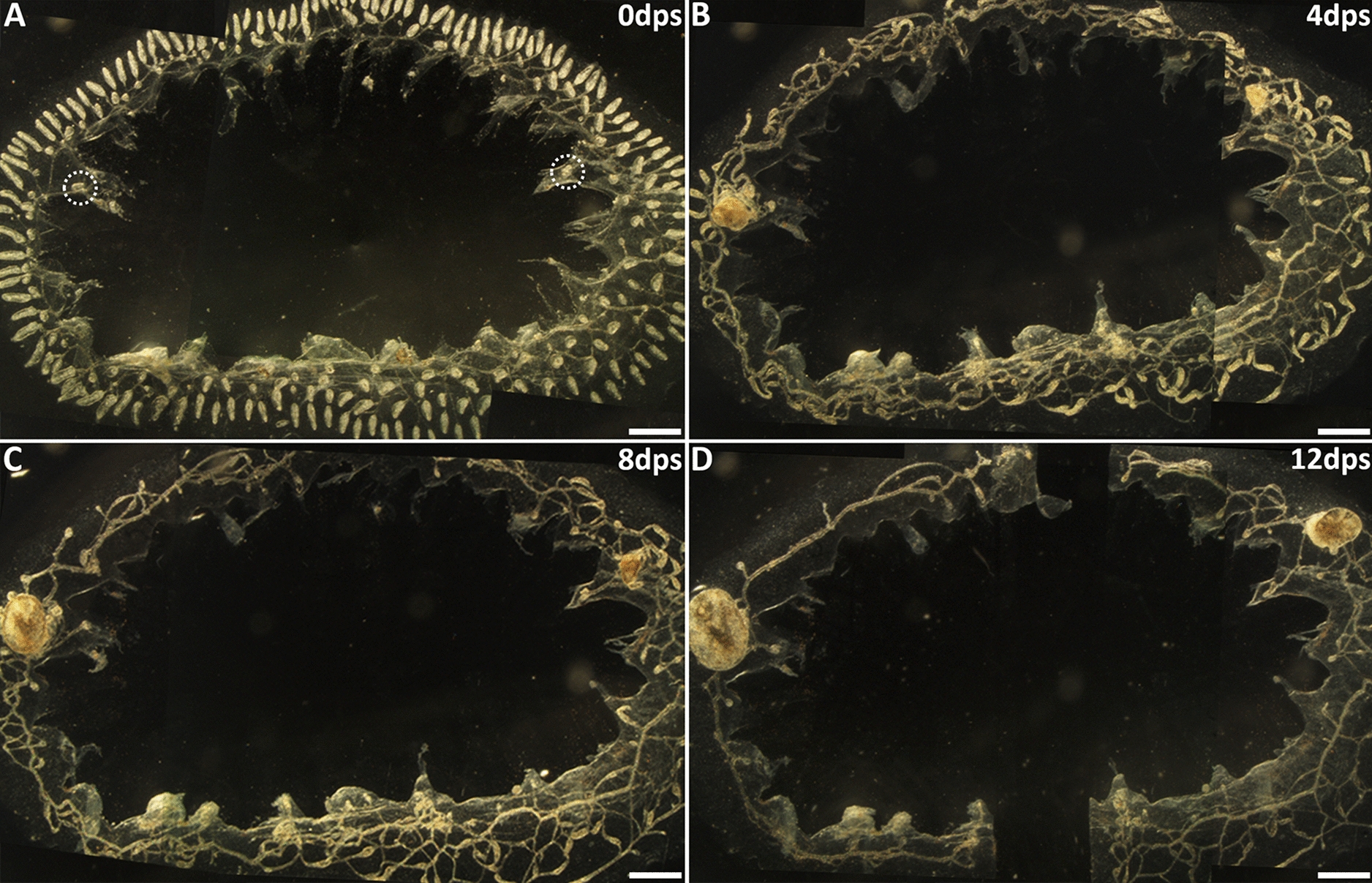
Secondary bud resorption is reversed after blood-borne crosstalk severed. **A** Post-surgery darkfield image of vasculature with two secondary buds (white dashed circles) connected through the blood vasculature. **B** At 4 days post-surgery, both secondary buds had fused to the common vasculature and continued development. **C** On day 8, the left secondary bud was identified as the purported winner because the right secondary bud had ceased growth. After image was taken, a section of blood vessels between secondary buds was removed. **D** By day 12 the loser bud had opened a siphon and began asexually budding. *dps* days post-surgery. Scale bars = 1.0 mm

To characterize the timing and mechanisms of competition, the circulation was severed following visual changes in growth rates between the two buds. In these experiments, colonies with two secondary buds (Fig. 9B) were left to develop following completion of heart development, and until one bud (presumed to be the winner) was growing steadily, and the other (presumed to be the loser) was not increasing in size. By day 8 we observed that the smaller secondary bud had started shrinking, indicating it was beginning to die (Fig. 9C, right side), and at that point the vasculature was severed. Within 4 days, the loser secondary bud had substantially increased in size, opened its siphons, and started to bud (Fig. 9D, right side).

**Fig. 10:**
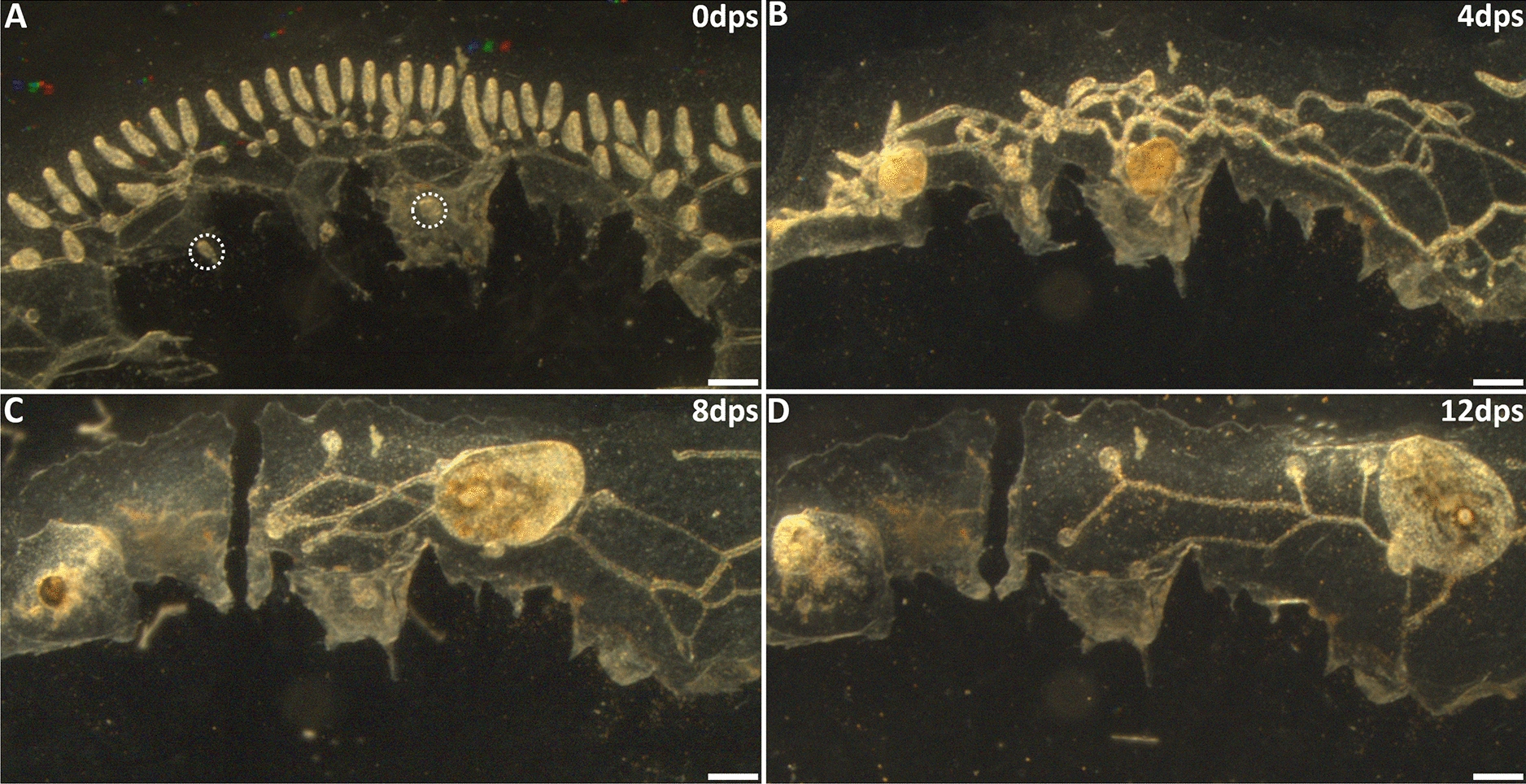
Loser bud resorption reversed at late stage of separation from winner bud. **A** Post-surgery darkfield image of vasculature and two secondary buds (white dashed circles). **B** Both secondary buds developed to a proportional size by day four post-surgery. **C** Blood vessels between zooids were severed 24 h prior to imaging. Loser secondary bud showed heavy pigmentation and was greatly reduced in size. **D** Five days after separation, the loser secondary bud had increased in size and opened a siphon. *dps* days post-surgery. Scale bars = 0.5 mm

We repeated this experiment, but this time allowed the smaller bud to decrease in size to a point where we could observe accumulation of pigmented cells in the body, which is characteristic of the later stages of apoptosis and phagocyte resorption, and at that point severed the vascular connection between them (Fig. 10). During the next few days, the loser bud increased in size, decreased in pigmentation, and eventually opened its siphons. These data indicate that although both buds have the potential to develop, signaling from a winner secondary bud creates a continuously repressive environment for the loser. Importantly, this also shows that a partially resorbed bud can reverse its fate and complete development.

In summary, both WBR in *B. diegensis* [24] and bud competition in *B. schlosseri* clearly show that a botryllid colony can shift metabolic resources within an individual to support development of a feeding zooid. In *B. schlosseri*, remnants of the peribranchial budding process can detect and respond to injury via migrating and reconnecting to the vasculature. If multiple developing zooids are attached to the common vascular network, a competitive situation arises in which only a single zooid completes development. This competition likely exists to increase the chances that a single bud reaches maturity. Afterward, the colony can feed and resume normal peribranchial budding.

## Discussion and conclusions

We began these experiments to understand why the size, circulation, and timing requirements for successful WBR would be so different between *B. schlosseri* and *B. diegensis*. Initially, we wanted to compare the cells responsible for WBR in *B. schlosseri* and *B. diegensis* using a rescue assay utilized in *B. diegensis* [24]. However, our results in *B. schlosseri* were inconsistent. We backtracked and carried out control experiments, following published protocols [14, 25, 33, 34], but could not repeat previous results. While we did see what appeared to be WBR several times, retrospective analyses of time lapse videos revealed that the source was always a piece of tissue inadvertently left following the ablation surgery which we observed migrating, reconnecting with the vasculature, then developing into a functional zooid. The ability of isolated and transplanted secondary buds to complete development has been described previously and is robust; thus, these results were not surprising [14]. We carried out controlled experiments in which portions of buds, including those purposefully damaged or partially resorbed, were left behind. These remaining tissues were always the source of the new zooid, and these experiments replicated every result that has been previously published for WBR in *B. schlosseri*; from the time to development, including the appearance of the heartbeat (6–12 days); to the presence of a phenotypically abnormal zooid in the first generation [14, 25, 33, 34] (Additional file 19: Table S4).

The simplest explanation of these results is that there are genetic or environmental differences between our local *B. schlosseri* population and those used in other studies. *B. schlosseri* is an introduced species to California, and there could have been a genetic bottleneck in the founding population, or environmental differences, and the ability to undergo WBR has been lost or is never triggered. In addition, as described above, there is plasticity to the use of peribranchial and vascular budding among the botrydallid species. Conversely, while one publication showed immunofluorescence images of aggregated cells and blastula-like structures [33], there are no clear longitudinal studies of WBR in *B. schlosseri*, such as a video showing bud development within the transparent vasculature. In addition, in our studies on *B. schlosseri* we found no evidence of these early stages, including using DAPI staining or expression of pluripotency markers (Additional file 9: Fig. S4), which we could use to easily visualize these structures in *B. diegensis* [24]. Moreover, if WBR is a survival strategy that has evolved for colony survival, the requirements for successful zooid development—a large colony, continued circulation, and will only regenerate a functional zooid during a 36-h window each week—seem unlikely to be strongly selected for. Yet it is those requirements that are consistent among different populations that have been studied [14, 25, 33, 34]. While we cannot prove a negative result, our data suggests that the unique requirements for *B. schlosseri* likely exist to increase the probability that ectopic development of a peribranchial bud will occur.

If our conclusions are correct, why would *B. diegensis* undergo WBR, while *B. schlosseri* does not? There are two interrelated hypotheses. First, it could be due to properties of the circulatory cells in each species. We recently found that the source of WBR in *B. diegensis* was a population of mobile cells expressing integrin-alpha-6 (IA6) [24]. In *B. schlosseri*, we have found that IA6+ cells are germline precursors [24, 35]. While we have not functionally established that IA6 + cells in *B. diegensis* are also lineage-restricted germline precursors—as this species is not reliably fertile in the lab—at a population level IA6+ cells in both species have highly similar expression patterns, including almost exclusive expression of both germline and pluripotency markers, such as pou3 [24, 35]. We are currently characterizing each population at single cell resolution but have hypothesized that in *B. diegensis* either the IA6+ cells are heterogeneous, containing both germline and somatic progenitors, or that the loss of zooids and buds triggers germline progenitors to reprogram to a somatic progenitor, analogous to teratoma formation. The amount of IA6 cells is not noticeably different between the two species (Additional file 9: Fig. S4) [24, 36]; thus, either the cell populations are different, and *B. diegensis* retains a mobile pluripotent somatic progenitor, or there is not an appropriate signal or niche for germline precursors to transdifferentiate in *B. schlosseri*.

Vascular budding also occurs when colonies are exiting dormancy, and these studies provide some insight into niche formation. Previous studies have utilized *B. leachii*, where the entry and exit of dormancy due to seasonal fluctuation in temperature has been thoroughly documented [13, 31, 32], and we can easily shift *B. diegensis* in and out of dormancy in the lab using changes in temperature (not shown). In addition, several other botryllids can undergo dormancy, including *Botrylloides lentus*, *B. delicatus*, *B. prealongus* and *B. crystallinus*. In all these species, the entry into dormancy involves resorption of all zooids and buds followed by vascular remodeling into a densely packed vascular mat, a process visually equivalent to WBR in *B. diegensis* shown here [22, 37–39]. Histological sections of the resulting vascular mats during dormancy in *B. leachii* [31] (weeks to months) following the resorption event reveal the presence of cell aggregates and blastula-like structures; the first two steps in WBR that appear to be the dormant state of vascular buds. While WBR has only been documented in *B. diegensis* (this study), *B. violaceous* [13, 23] and *B. leachii* [28, 31], there appears to be a link between exiting dormancy and robust WBR, and that could be due to formation of a vascular niche. In contrast, there are neither published reports of dormancy in *B. schlosseri*, nor have we ever seen any process that resembles the entrance to dormancy in our lab.

**Fig. 11:**
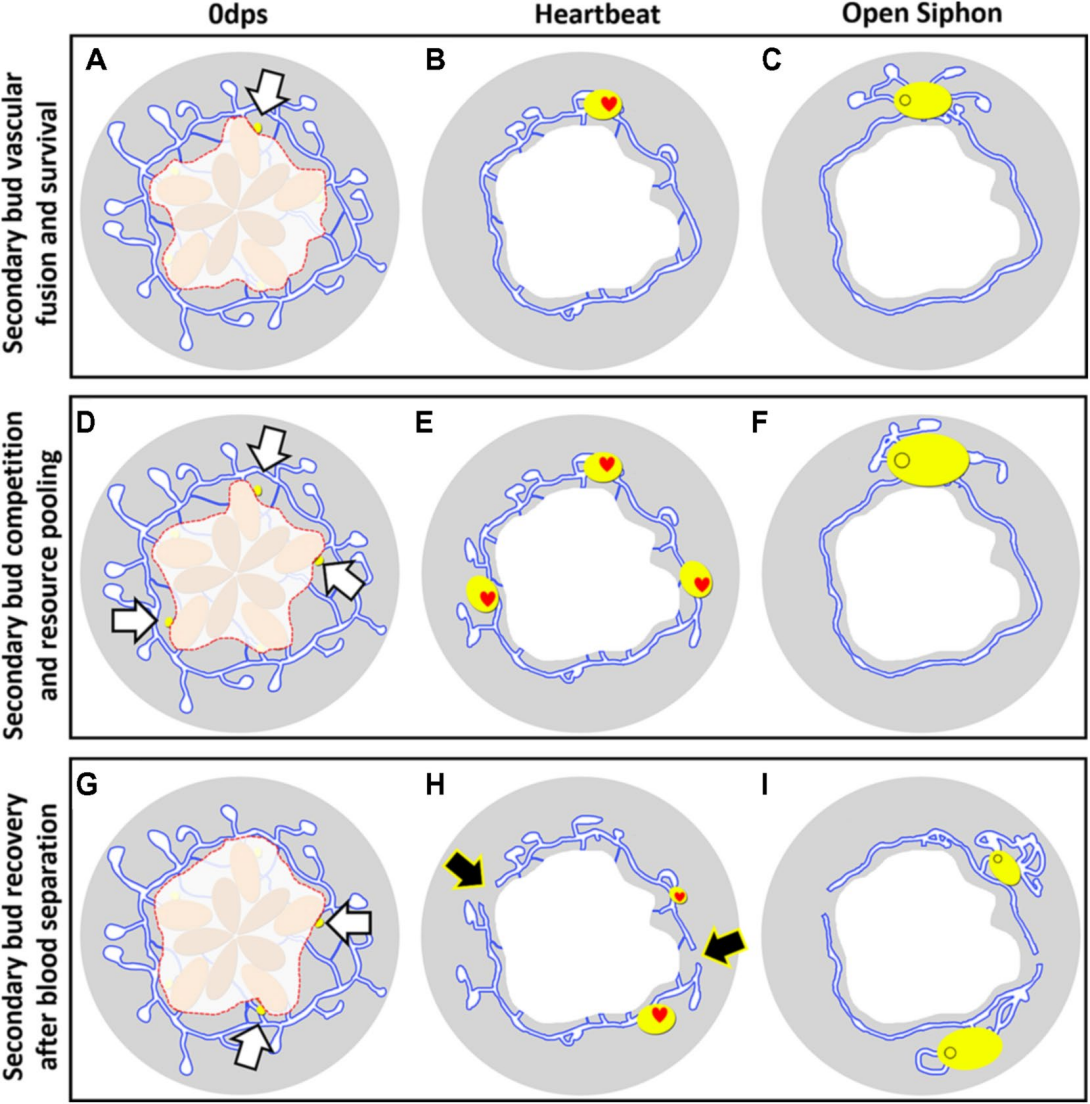
Scenarios of post-surgery secondary bud development in *Botryllus schlosseri*. **A** Illustration showing surgical isolation of a single secondary bud (white arrow). **B** By day 6, the vasculature is seen coalescing around the secondary bud, and a beating heart is observed. **C** The siphon opens on average by day 12 post-surgery and most of the vasculature collapses except for the area surrounding the newly developed zooid. **D** Illustration showing surgery performed to isolate three secondary buds. **E** All secondary buds form heartbeats even if they share the same blood vasculature. **F** Only one zooid opens a siphon. Other secondary buds are resorbed into the blood vasculature and provide extra growth to the remaining zooid. **G** Illustration showing surgery performed isolate two secondary buds. **H** Secondary buds are separated from blood communication (black arrows). **I** Two zooids are present because blood-borne competition was inhibited, and prospective loser bud was capable of full development

The major difference between *B. schlosseri* and *B. diegensis* is the response of the vasculature to zooid and bud ablation (Fig. 2). The *B. diegensis* vasculature remodels into a large mat, which is the exact same phenotype as the response to surgery and dormancy in all botryllid species in which either of these have been documented. In contrast, in *B. schlosseri*, the vasculature undergoes a very typical regression then expansion, maintaining the anatomy and robust blood flow via pumping of the ampullae (Additional file 5: Video S3), and if just a section of vasculature is cut off, it dies (Figs. 2, 5A–F). Thus, it may be that *B. schlosseri* does not make a niche for WBR to occur. However, given this divergent response, it is intriguing that a secondary bud responds to surgical separation via migrating away from its original location and reassociating with the colonial vasculature. We found that reassociation was necessary for further development; and equivalent conclusions were made in previous experiments where only the zooids were ablated, leaving both primary and secondary buds [14]. It is difficult to believe this is a coincidence and suggests that ectopic development of leftover peribranchial buds is the response to injury (Fig. 11). The fact that a zooid can develop from a secondary bud and only a small amount of vasculature, and even from a partially resorbed primary bud, further support this hypothesis (Figs. 5, 10).

The seemingly widespread vascular budding ability within the botryllids, along with the report of vascular budding in a species outside of the Botryllinae, *Symplegma brakenhielmi* [11], would suggest that WBR evolved prior to the origin of the botryllids and was subsequently lost in various lineages or has evolved convergently multiple times. Both of these two hypotheses fit with the known plasticity of budding modes within Styelidae [16]. However, careful observations of budding in *Symplegma reptans* and *Symplegma viride* show that all buds arise from the peribranchial epithelium in these species, but lose connection with the adult at a very early stage and migrate within the tunic toward the colony periphery [10, 40, 41]. It is possible that precocious peribranchial bud detachment occurs in *S. brakenhielmi* as well, such that buds appear to arise in the vasculature [11]. Early peribranchial bud detachment was also noted in *Botryllus tyreus* [42], which molecular phylogenies place as an early branching botryllid [43, 44], further supporting a third hypothesis—that peribranchial bud detachment and migration rather than vascular budding is ancestral for botryllids. If WBR is truly absent in *B. schlosseri*, as our study suggests, and in *S. brakenhielmi* (which is simply a speculation), that reduces the taxonomic breadth of WBR. The remaining species documented to have vascular budding, with currently well-resolved phylogenetic positions, all fall within a single clade, notably to the exclusion of *B. schlosseri* [45, 46]. Thus, if the third hypothesis is correct, vascular budding and WBR evolved once within one clade of the Botryllinae and appear to be predominantly linked to coping with environmental adversity—either for survival after major injury or as a mechanism to allow for colony dormancy and future recovery. Further, if this inference is correct, it suggests that *B. schlosseri* did not lose WBR, but that this capacity was gained in a separate clade. It is also interesting to note that precocious dissociation and migration of peribranchial buds has been a source of controversy in studies on vascular budding for over a century. In early descriptions of *B. schlosseri*, buds were reported to arise from the vasculature [47, 48]. Later researchers disputed this claim [49–52]. With another report of vascular budding in a different botryllid species [53], the dispute was reignited. However, further studies of *B. schlosseri* continued to contest claims of vascular budding [54–56]. The disputers suggested that the error was due to early bud migration [57], poorly conserved specimens [54], and other artifacts, that made peribranchial buds appear to have arisen vascularly.

Finally, one characteristic that is shared between *B. schlosseri* and *B. diegensis* is the competition between developing buds following injury, resulting in the development of only a single zooid. This was also documented in *B. leachii* [26], suggesting this competition is a quality control process which ensures that a single, healthy zooid develops which can regenerate an entire colony. It is interesting to note that in both *B. schlosseri* (this study) and *B. leachii* [26], the minimum amount of biomass to support zooid development has been defined, yet competition occurs even when the amount of biomass is well above that minimum (Fig. 5). In other words, only one zooid completes development even though there is enough vasculature to theoretically support development of multiple individuals. In terms of resources and growth, it is also intriguing that the presence of a loser bud causes the winner to be larger when it completes development, whereas the presence of a larger vascular network does not. While the mechanisms underlying catabolism and re-allocation or resources from these tissues to the developing buds are unknown, we have found that this competition is mediated by cells or circulatory factors which can suppress bud development in a reversible manner (Figs. 8, 9, 10). Unlike the *Botrylloides* species, which form a large, opaque vascular mat following surgery, the *B. schlosseri* vasculature retains its anatomy. This gives us spatial and temporal control of the circulatory link between developing buds, and provides a powerful new model to study the cellular and molecular mechanisms underlying resource allocation and competition within an individual.

## Methods

### Animal husbandry

Colonies of *Botryllus schlosseri* and *Botrylloides diegensis* were collected from Santa Barbara Marina in California, USA (GPS coordinates 34° 24′ 24″ N/119° 41′ 25″ W). Separate genotypes of *B. schlosseri* were distinguished by their color morphology. If the same color morphology was collected, then samples were obtained from distant dock slips to ensure genotypic variety. Hatches were collected on glass slides and raised in a mariculture room with constant flowing seawater and temperature ranging from 19° to 21 °C. They were fed microalgae every hour, and glass slides were cleaned of parasites every 2 weeks using Kim-wipes and soft synthetic bristle paint brushes (size number 2).

### Surgeries

*Botryllus schlosseri:* Removal of tissue was performed using the following tools: fine forceps (Dumostar/55), micro-surgery scissors (FST/15400-12), and razor blades (Personna/0.009RD). Surgeries were done under a stereomicroscope (Zeiss Stemis 2000) at magnifications between 40× and 50×. Animals were cleaned using a round-tip paint brush a day prior to surgery to reduce negative effects from parasitism, and subsequently left to develop in stagnant 0.5 µm filtered seawater. When a feeding siphon opened, animals were transferred to mariculture tanks.

*Botrylloides diegensis*: Separation of zooids from blood vasculature was accomplished using a razor blade (Personna/0.009RD). Animals were transferred to a new slide, and vasculature regenerated while adhered to original slide in 0.5 µm filtered seawater at temps ranging from 19° to 21 °C.

#### To remove all zooids and developing buds from *Botryllus schlosseri*

Zooids, primary buds, and secondary buds were cut out using micro-surgery scissors. All bodies were then removed at once using fine forceps. A paintbrush was then used to remove unwanted debris left after surgery. Blood restoration and circulation in colony vasculature was assessed under a dissecting microscope in our mariculture facility.

#### To remove all zooids, and leave an anterior primary bud fragment with secondary bud in *Botryllus schlosseri*

Zooids, and all but one anterior primary bud and secondary bud were cut out using micro-surgery scissors. Bodies were then removed at once using fine forceps. A paintbrush was used to remove unwanted debris left after surgery.

#### To remove all zooids, and leave only a developing secondary bud in *Botryllus schlosseri*

Tissue was excised using a new razor blade and fine forceps. A cut was made to gape the excurrent siphon region, and resorbing zooids were teased out of the surgical hole using forceps. The primary buds were then opened down the middle from the anterior-most point, and the two sections were slowly pulled out using forceps while being careful not to pull or damage the secondary bud.

### Microscopy

Micrographs of animals and tissues were acquired using a Leica MZ16 FA stereoscope with Q-imaging Retiga digital CCD camera on Image-Pro MDA software. Regeneration and competition assays were imaged daily and then placed back into mariculture overnight. Timelapses were maintained under the microscope in a temperature-controlled basin at 19 °C with circulating filtered seawater. Timelapse image settings used: 80 ms exposure with one image taken every five minutes. Image sets were formatted into AVI files using ImageJ [58].

### Data analysis

Wilcoxon signed-rank test was used to determine statistical significance between groups of data with unequal variance for Figs. 4 and 8.

## Supplementary Information

**Additional file 1: Figure S1.** Asexual budding cycle in *Botryllus schlosseri*. **A** Darkfield image of 15-zooid colony. The blue dashed lines demarcate an extracorporeal vasculature that allows for shared blood flow amongst the colony. Zooids (white dashed lines) and a developing primary bud (white arrow) grow concurrently. **B** Colony in panel A after 6 days. Zooids undergo takeover and are replaced by the primary buds (white arrow). Also shown is a third generation, the secondary bud (black arrow), growing directly from the primary bud epithelium. Panels C through G show intermediate stages. **C** During stage A1, the zooid’s siphon opens, the primary bud is visible (white arrow), and the secondary bud is nascent. **D** At stage B1, the secondary bud (black arrow) has formed a double vesicle. **E** Stage C is where organogenesis is occurring in the secondary bud (black arrow). **F** Stage D is takeover, where zooids are resorbed and replaced by the subsequent generation. **G** After 7 days, what was initially the primary bud, is now a filter-feeding zooid (white arrow) with an open siphon. The secondary bud has developed into the primary bud (black arrow), and the process repeats. **H**–**L** Illustrations following blastogenesis in C–G, respectively. Scale bars = 0.5 mm.

**Additional file 2: Video S1.** Blastogenetic cycle of *Botryllus schlosseri*. A weeklong timelapse showing the asexual budding cycle starting at stage A and proceeding through takeover until reaching stage A again.

**Additional file 3: Figure S2.** Marginal blood vessel demarcation in *Botryllus schlosseri*. Prior to surgery, this blood vessel interconnects all zooids and developing bodies within in a system of zooids. Directly after surgery, the marginal vessel (indicated by white arrows) was damaged due to proximity of secondary buds. While removal of all secondary buds causes damage to this blood vessel, a new marginal vessel was restored 24 h after surgery. Scale bar = 0.5 mm.

**Additional file 4: Video S2.** Marginal vessel blood flow. 24 h after surgery, any damage to the marginal vessel has been repaired and blood flow is vigorous around the entire colony.

**Additional file 5: Video S3.** Isolation of and subsequent rearrangement of vasculature. A 5-day timelapse showing the activity of vasculature after removal of all zooids, primary buds, and secondary buds.

**Additional file 6: Video S4.** Secondary bud surviving surgical assay, migrating, and continuing development. A timelapse showing the activity of a secondary bud after attempts to induce whole body regeneration by removing all zooids and developing bodies.

**Additional file 7: Figure S3.** Experiments to induce whole body regeneration in *Botryllus schlosseri*. **A**–**C** Darkfield images of post-surgery colonies of *B. schlosseri* at day 0. Zooids and all developing buds were removed. **D**–**F** Same systems shown in panels A–C, respectively, at day 9 post-surgery. Zero colonies regenerated a zooid (*n* = 128). dps = days post-surgery. Scale bars = 0.5 mm.

**Additional file 8: Table S1.** Whole body regeneration potential between different genotypes. To assess whole body regeneration (WBR) capability across genotypes, multiple individual colonies strains were examined. We performed from 5 to 89 surgeries on each genotype, but no vasculature gave indication for a WBR event. Animals were collected at the Santa Barbara Marina in California.

**Additional file 9: Figure S4.** Circulatory cell dynamics following ablation surgery in *B. schlosseri*. Panels A–F show the response of pluripotent and mitotically active circulatory cells for 96 h. following ablation surgery. Cells are labeled by in situ hybridization for expression of the pluripotency marker pou3 [24, 36], and counterstained with a marker for mitosis (an antibody for phosphohistone H3) and the DNA stain, DAPI. While mitotically active pluripotent cells were observed, no cellular aggregations or other developmental structures (e.g., a double vesicle) were present. In contrast, both cellular aggregations and vesicular structures could be easily seen within 48 h in *B. diegensis* using equivalent probes (see reference [24] for comparison and experimental methods, the latter are equivalent for both species). hps = hours post-surgery. Blue (DAPI) = nuclei, Green (phosphohistone H3) = dividing cells, red (pou3) = putative stem cell marker. Scale bars = 0.5 mm.

**Additional file 10: Figure S5.** Large colony surgery to increase chances of inducing whole body regeneration. **A** Post-surgery darkfield image of a five-system colony. There were approximately 250 ampullae all connected by a ring of vasculature with vigorous blood flow. Animal was maintained in filtered seawater and no evidence of a developing bud was detected. By day 12 the blood flow had ceased, hyper-pigmentation was present, and tissue movement had halted. dps = days post-surgery. Scale bar = 0.5 mm.

**Additional file 11: Figure S6.** Post-surgery secondary bud development with anterior primary bud fragments. **A** Illustration showing surgery performed at stage B1 to isolate anterior half of the primary bud with secondary bud. **B**, **C** Darkfield images of post-surgery B1 colony at day 0 and 7, respectively. **D** Illustration showing surgery performed at stage C1 to isolate the anterior half of the primary bud with secondary bud. **E**, **F** Darkfield images of post-surgery C1 colony at day 0 and 7, respectively. **G** Illustration showing surgery performed at stage D (takeover) to isolate the anterior half of the primary bud with secondary bud. **H**, **I** Darkfield images of post-surgery D1 colony at day 0 and 7, respectively. **J** Quantitative analysis of post-surgery zooid development with bud tissues left behind at stages B–D of the asexual life cycle. Zooids developed in 70%, 86%, and 97% of surgeries when performed in stages B, C, and D, respectively. dps = days post-surgery. Scale bars = 1 mm.

**Additional file 12: Figure S7.** Abnormal first generation from isolated secondary buds are fully developed with open siphon. **A** Secondary bud developed with abnormal morphology. Animals are commonly shown with dorsal side up and oral siphon visible from above, but here the siphon is pointing toward the right side (white arrow). **B** Secondary bud developmental duplication with two oral siphons (white arrows) and a single atrial siphon (black arrow). Scale bars = 0.5 mm.

**Additional file 13: Table S2.** Raw data for Fig. 4. Measured values used to create box plot shown in Fig. 4. Animals were checked once a day under a dissecting microscope for developmental progression.

**Additional file 14: Figure S8.** Assessing the potential for whole body regeneration from damaged secondary bud. **A** Post-surgery darkfield image of colony of *B. schlosseri* with a single secondary bud left behind that has been fragmented through the application of external pressure via forceps. **B** Magnified view of fragmented secondary bud from panel A. **C** Secondary bud developed a heartbeat at day 7. **D** Secondary bud resorbs by day 14, blood flow has ceased, and the vasculature has collapsed. dps = days post-surgery. Scale bars = 0.5 mm.

**Additional file 15: Video S5.** Secondary bud migration. This timelapse shows the hours directly following surgery and how secondary buds migrate away from their original position toward the vasculature.

**Additional file 16: Figure S9.** WBR from large vascular beds in *Botrylloides diegensis.* **A**–**C** A single zooid regenerating from a relatively large patch of vasculature. **D**–**F** Solitary zooid regeneration from two patches of ampullae connected by a single blood vessel. dps = days post-surgery. Scale bars = 2 mm.

**Additional file 17: Table S3.** Raw data for Fig. 8. Measured values used to create box plot shown in Fig. 8. Area values collected using ImageJ software [58].

**Additional file 18: Figure S10.** Post-surgery secondary buds develop independently while sharing tunic but not blood in *Botryllus schlosseri*. **A** Darkfield image of two systems within the same colony. **B** One secondary bud was isolated from each system (white circles). Two systems were left partially connected via the tunic (black arrow); however, the blood vasculature was removed from the region between them. **C** By day 5 each bud had developed a beating heart. **D** Both buds developed into filter-feeding adults. dps = days post-surgery. Scale bars = 0.5 mm.

**Additional file 19: Table S4.** Summary of how secondary bud isolation events explain WBR observations. The characteristics and requirements for WBR in *Botryllus schlosseri* match six observations when only a single secondary bud is isolated with vascular tissues after removal of all zooids and other buds.

## Data Availability

Data generated and analyzed for this study are included in this published article (including all additional files).
