# Supplementary figures and images for: Whole body regeneration and developmental competition in two botryllid ascidians
Source: EvoDevo. 2021 Dec 15;12:15. doi: 10.1186/s13227-021-00185-y (PMC8675491; doi:10.1186/s13227-021-00185-y)

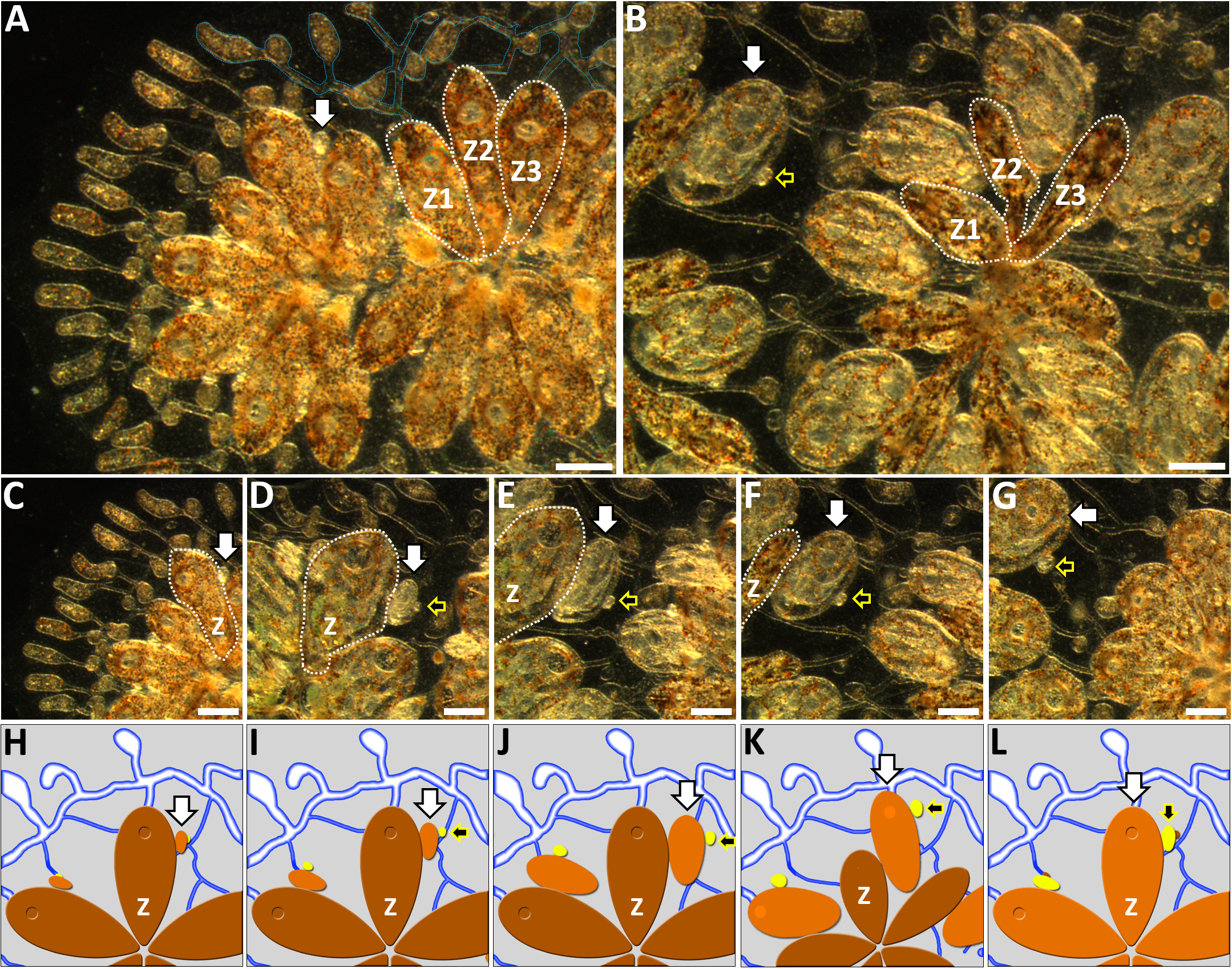

Supplement: Supplementary file 1 — Additional file 1: Figure S1. Asexual budding cycle in Botryllus schlosseri. A Darkfield image of 15-zooid colony. The blue dashed lines demarcate an extracorporeal vasculature that allows for shared blood flow amongst the colony. Zooids (white dashed lines) and a developing primary bud (white arrow) grow concurrently. B Colony in panel A after 6 days. Zooids undergo takeover and are replaced by the primary buds (white arrow). Also shown is a third generation, the secondary bud (black arrow), growing directly from the primary bud epithelium. Panels C through G show intermediate stages. C During stage A1, the zooid’s siphon opens, the primary bud is visible (white arrow), and the secondary bud is nascent. D At stage B1, the secondary bud (black arrow) has formed a double vesicle. E Stage C is where organogenesis is occurring in the secondary bud (black arrow). F Stage D is takeover, where zooids are resorbed and replaced by the subsequent generation. G After 7 days, what was initially the primary bud, is now a filter-feeding zooid (white arrow) with an open siphon. The secondary bud has developed into the primary bud (black arrow), and the process repeats. H–L Illustrations following blastogenesis in C–G, respectively. Scale bars = 0.5 mm. [file 13227_2021_185_MOESM1_ESM.tif]

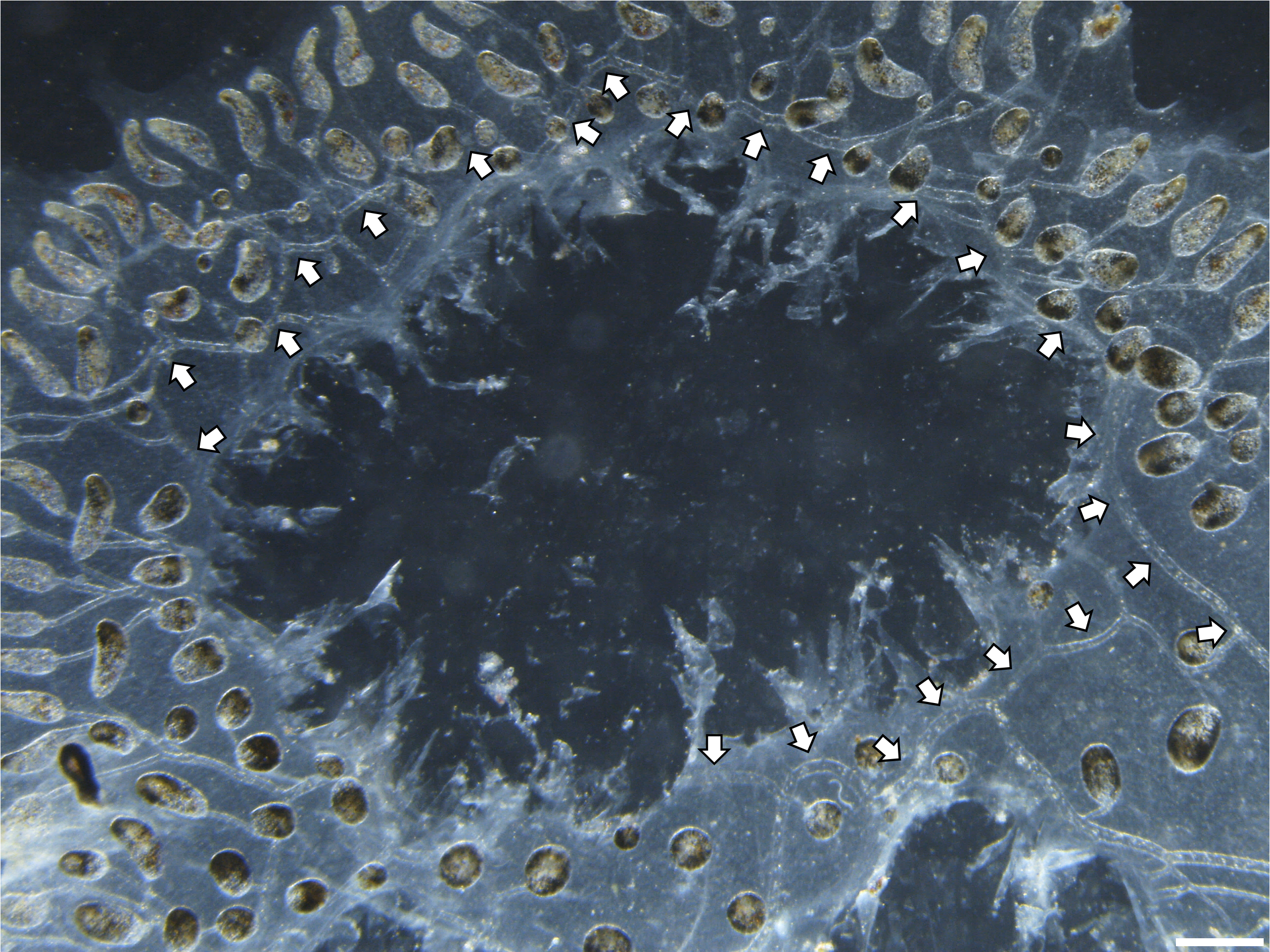

Supplement: Supplementary file 3 — Additional file 3: Figure S2. Marginal blood vessel demarcation in Botryllus schlosseri. Prior to surgery, this blood vessel interconnects all zooids and developing bodies within in a system of zooids. Directly after surgery, the marginal vessel (indicated by white arrows) was damaged due to proximity of secondary buds. While removal of all secondary buds causes damage to this blood vessel, a new marginal vessel was restored 24 h after surgery. Scale bar = 0.5 mm. [file 13227_2021_185_MOESM3_ESM.tif]

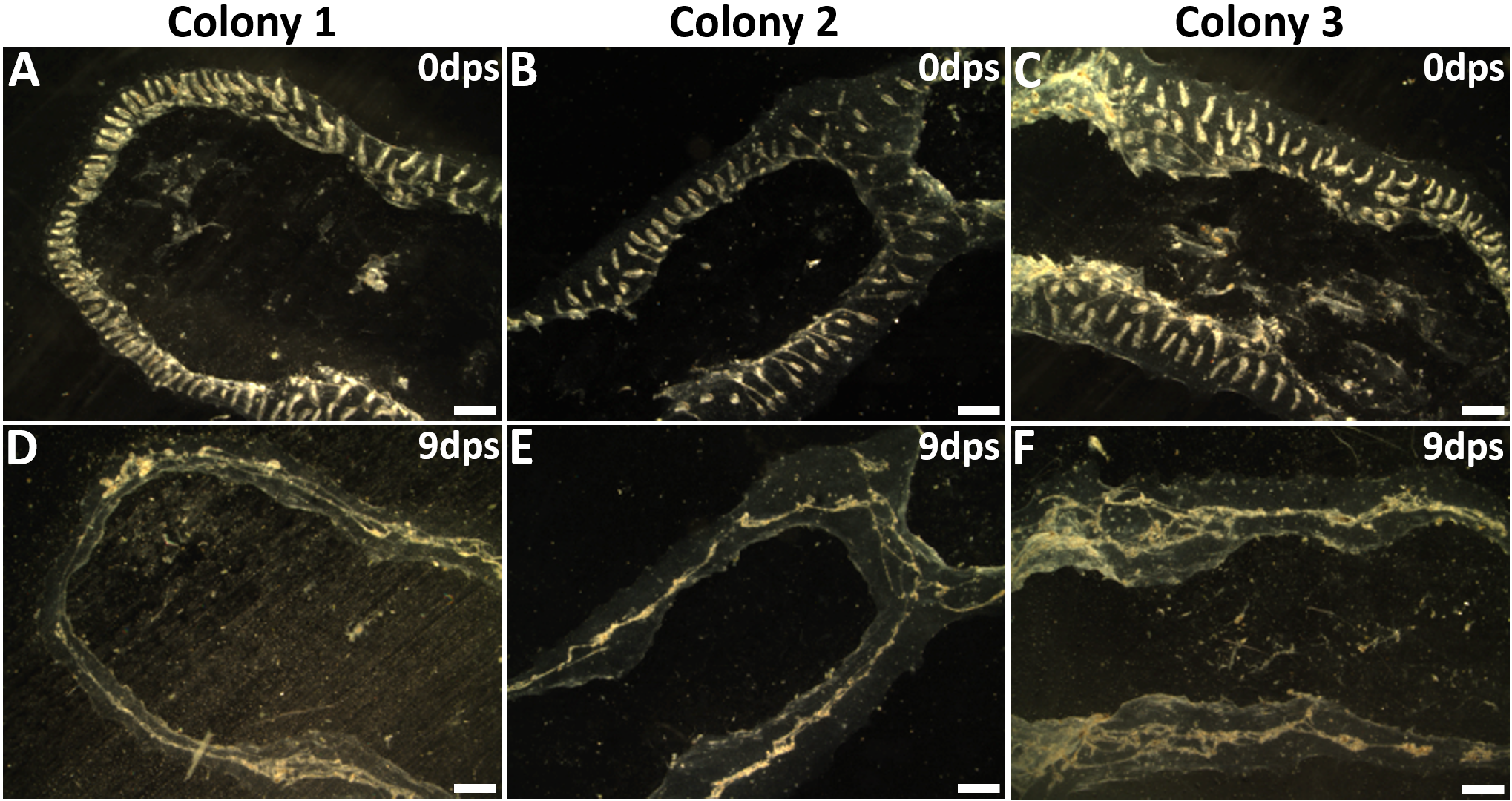

Supplement: Supplementary file 7 — Additional file 7: Figure S3. Experiments to induce whole body regeneration in Botryllus schlosseri. A–C Darkfield images of post-surgery colonies of B. schlosseri at day 0. Zooids and all developing buds were removed. D–F Same systems shown in panels A–C, respectively, at day 9 post-surgery. Zero colonies regenerated a zooid (n = 128). dps = days post-surgery. Scale bars = 0.5 mm. [file 13227_2021_185_MOESM7_ESM.tif]

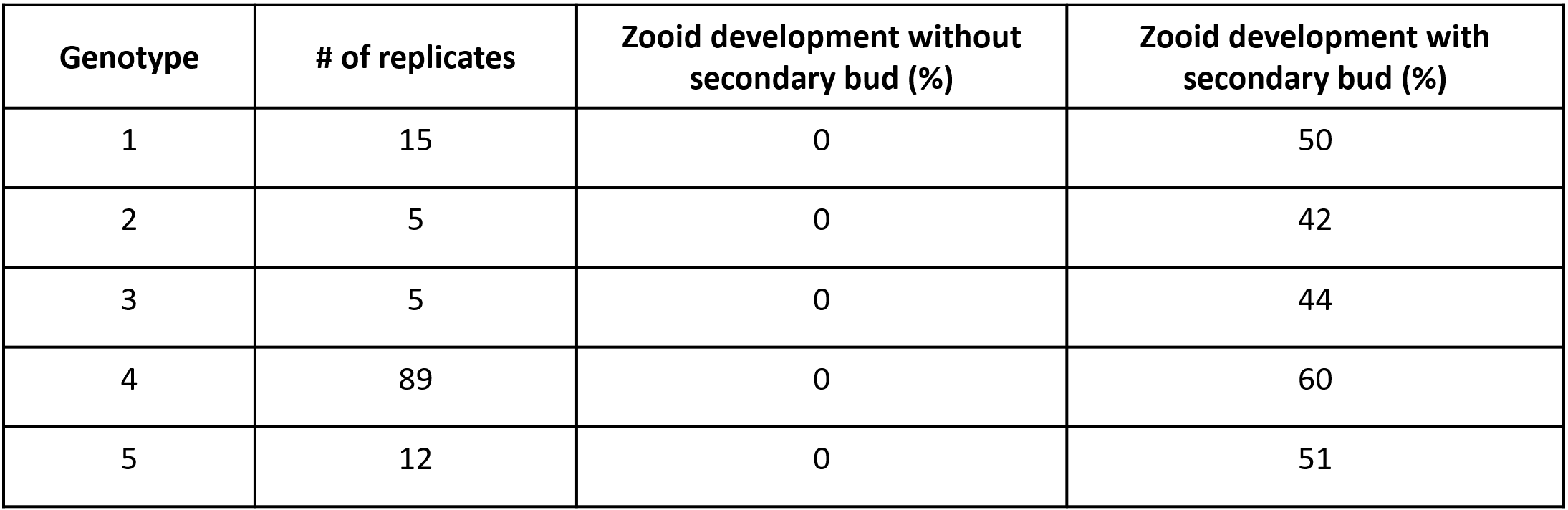

Supplement: Supplementary file 8 — Additional file 8: Table S1. Whole body regeneration potential between different genotypes. To assess whole body regeneration (WBR) capability across genotypes, multiple individual colonies strains were examined. We performed from 5 to 89 surgeries on each genotype, but no vasculature gave indication for a WBR event. Animals were collected at the Santa Barbara Marina in California. [file 13227_2021_185_MOESM8_ESM.tif]

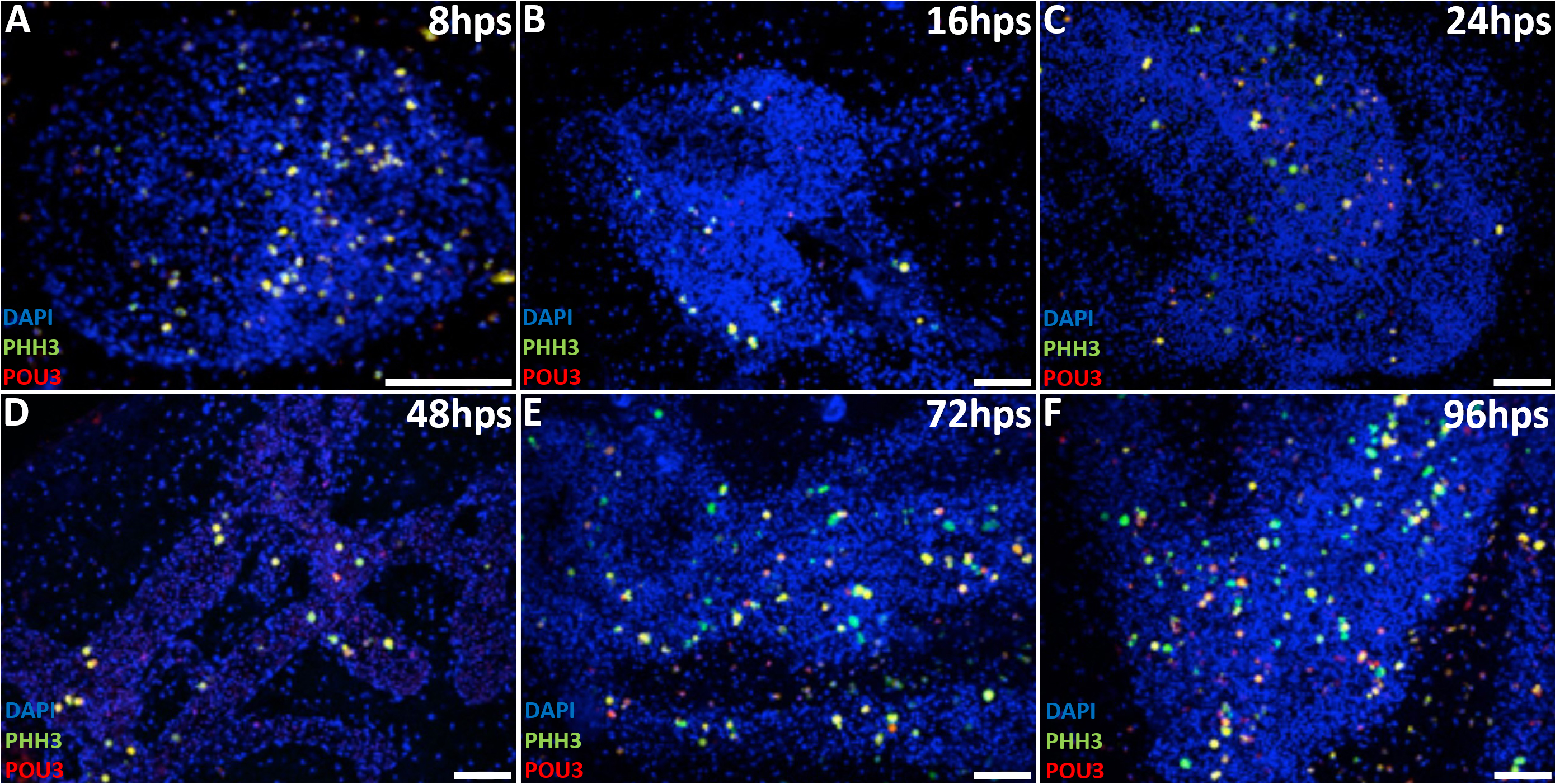

Supplement: Supplementary file 9 — Additional file 9: Figure S4. Circulatory cell dynamics following ablation surgery in B. schlosseri. Panels A–F show the response of pluripotent and mitotically active circulatory cells for 96 h. following ablation surgery. Cells are labeled by in situ hybridization for expression of the pluripotency marker pou3 [24, 36], and counterstained with a marker for mitosis (an antibody for phosphohistone H3) and the DNA stain, DAPI. While mitotically active pluripotent cells were observed, no cellular aggregations or other developmental structures (e.g., a double vesicle) were present. In contrast, both cellular aggregations and vesicular structures could be easily seen within 48 h in B. diegensis using equivalent probes (see reference [24] for comparison and experimental methods, the latter are equivalent for both species). hps = hours post-surgery. Blue (DAPI) = nuclei, Green (phosphohistone H3) = dividing cells, red (pou3) = putative stem cell marker. Scale bars = 0.5 mm. [file 13227_2021_185_MOESM9_ESM.tif]

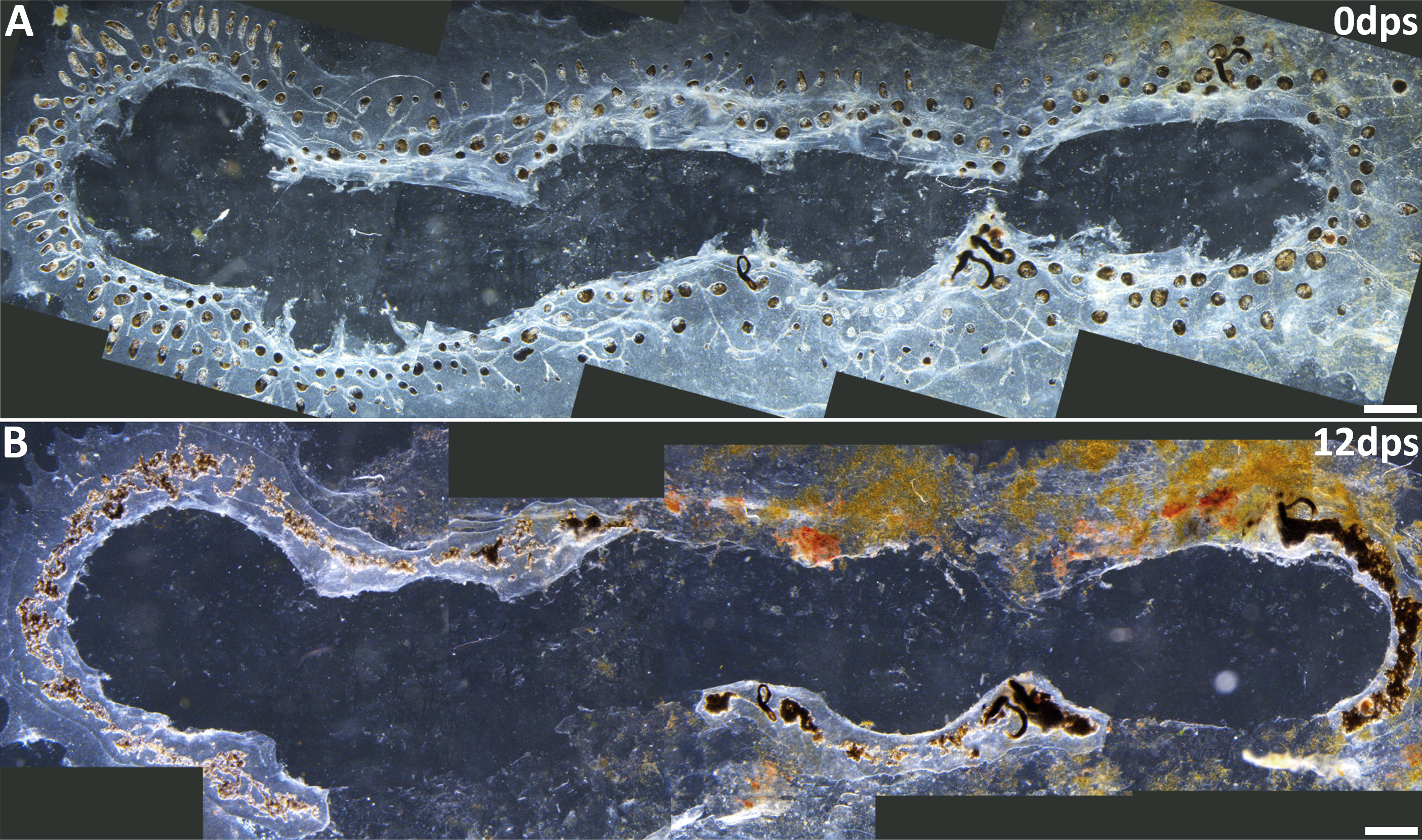

Supplement: Supplementary file 10 — Additional file 10: Figure S5. Large colony surgery to increase chances of inducing whole body regeneration. A Post-surgery darkfield image of a five-system colony. There were approximately 250 ampullae all connected by a ring of vasculature with vigorous blood flow. Animal was maintained in filtered seawater and no evidence of a developing bud was detected. By day 12 the blood flow had ceased, hyper-pigmentation was present, and tissue movement had halted. dps = days post-surgery. Scale bar = 0.5 mm. [file 13227_2021_185_MOESM10_ESM.tif]

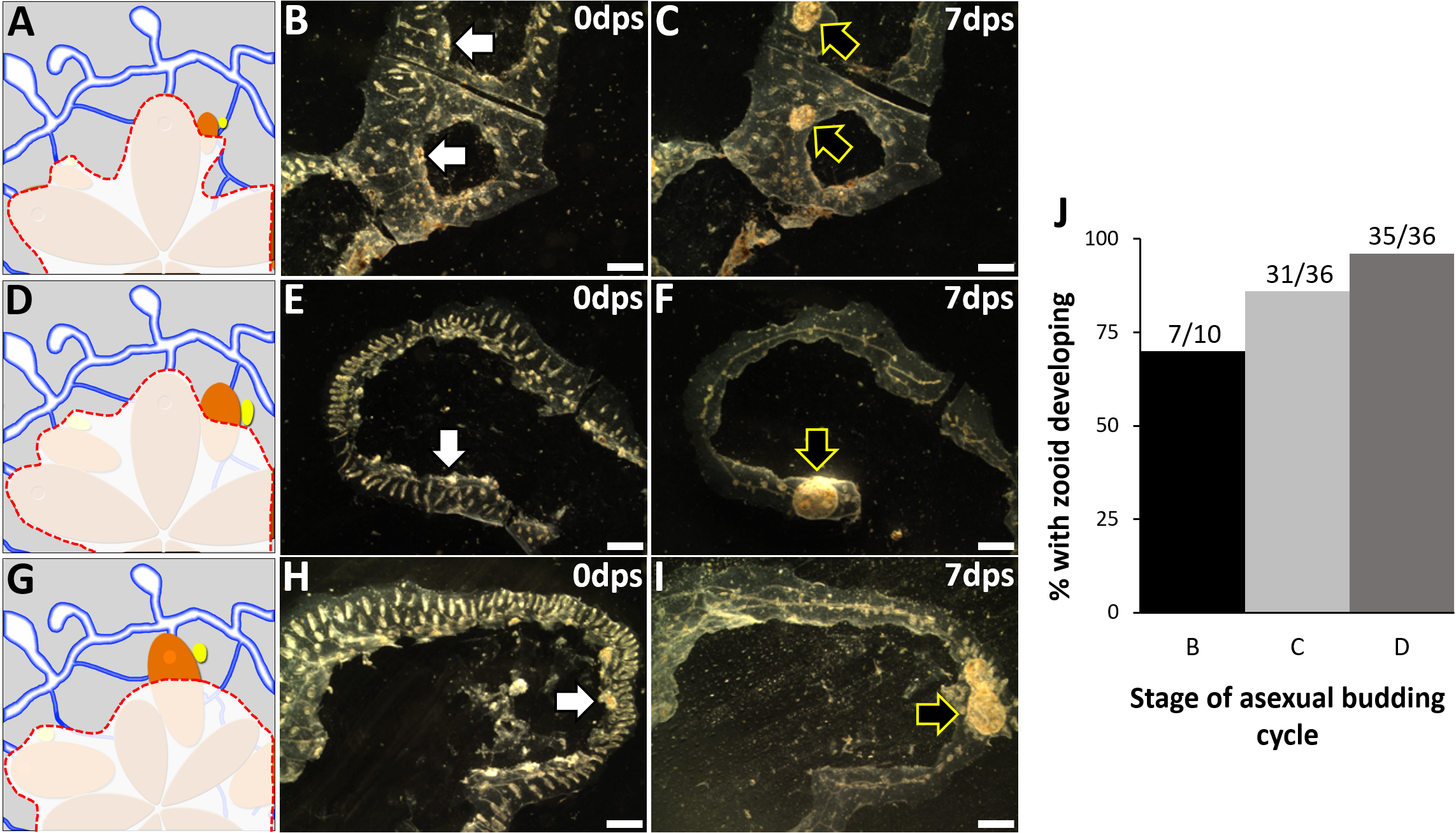

Supplement: Supplementary file 11 — Additional file 11: Figure S6. Post-surgery secondary bud development with anterior primary bud fragments. A Illustration showing surgery performed at stage B1 to isolate anterior half of the primary bud with secondary bud. B, C Darkfield images of post-surgery B1 colony at day 0 and 7, respectively. D Illustration showing surgery performed at stage C1 to isolate the anterior half of the primary bud with secondary bud. E, F Darkfield images of post-surgery C1 colony at day 0 and 7, respectively. G Illustration showing surgery performed at stage D (takeover) to isolate the anterior half of the primary bud with secondary bud. H, I Darkfield images of post-surgery D1 colony at day 0 and 7, respectively. J Quantitative analysis of post-surgery zooid development with bud tissues left behind at stages B–D of the asexual life cycle. Zooids developed in 70%, 86%, and 97% of surgeries when performed in stages B, C, and D, respectively. dps = days post-surgery. Scale bars = 1 mm. [file 13227_2021_185_MOESM11_ESM.tif]

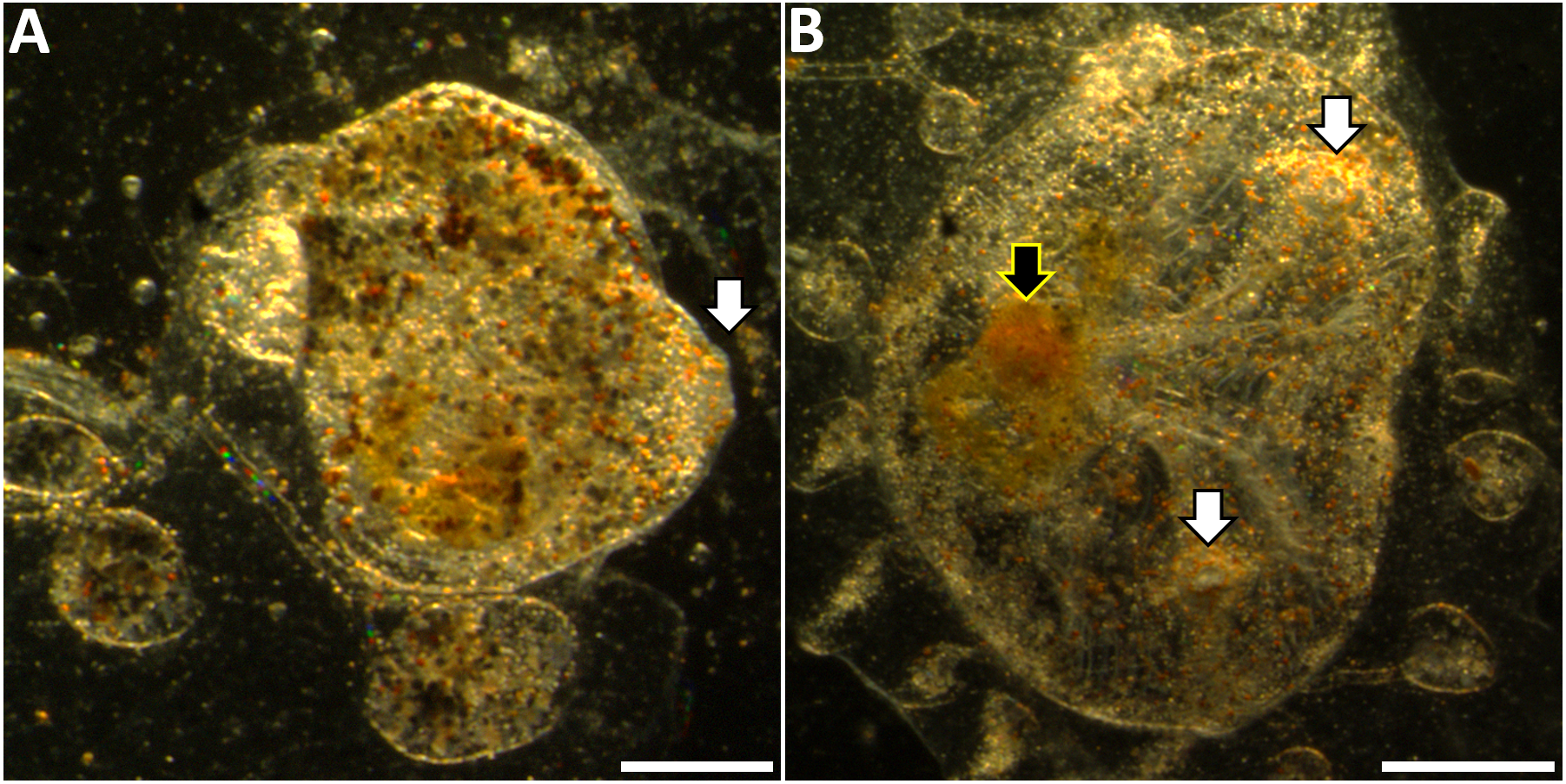

Supplement: Supplementary file 12 — Additional file 12: Figure S7. Abnormal first generation from isolated secondary buds are fully developed with open siphon. A Secondary bud developed with abnormal morphology. Animals are commonly shown with dorsal side up and oral siphon visible from above, but here the siphon is pointing toward the right side (white arrow). B Secondary bud developmental duplication with two oral siphons (white arrows) and a single atrial siphon (black arrow). Scale bars = 0.5 mm. [file 13227_2021_185_MOESM12_ESM.tif]

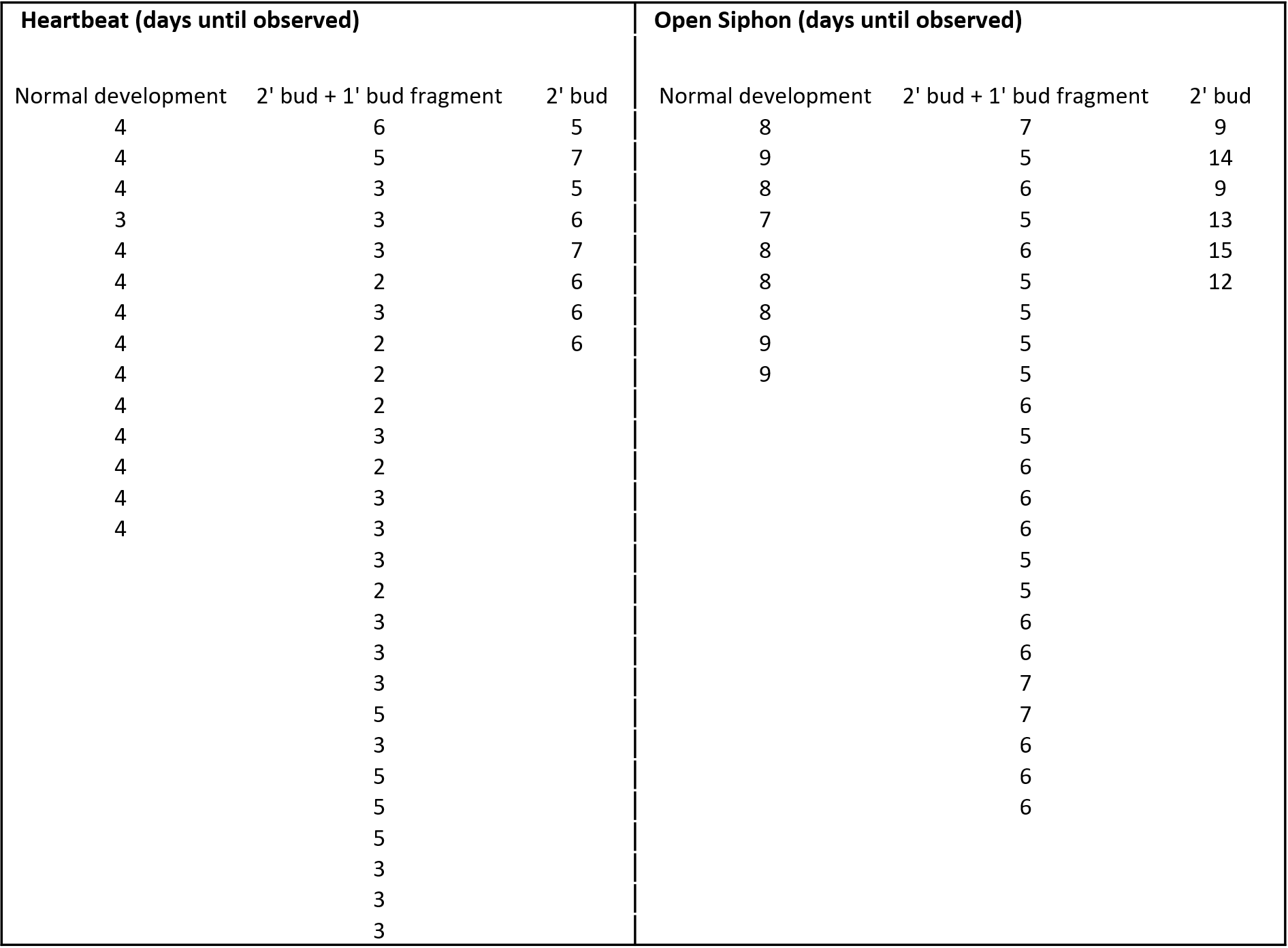

Supplement: Supplementary file 13 — Additional file 13: Table S2. Raw data for Fig. 4. Measured values used to create box plot shown in Fig. 4. Animals were checked once a day under a dissecting microscope for developmental progression. [file 13227_2021_185_MOESM13_ESM.tif]

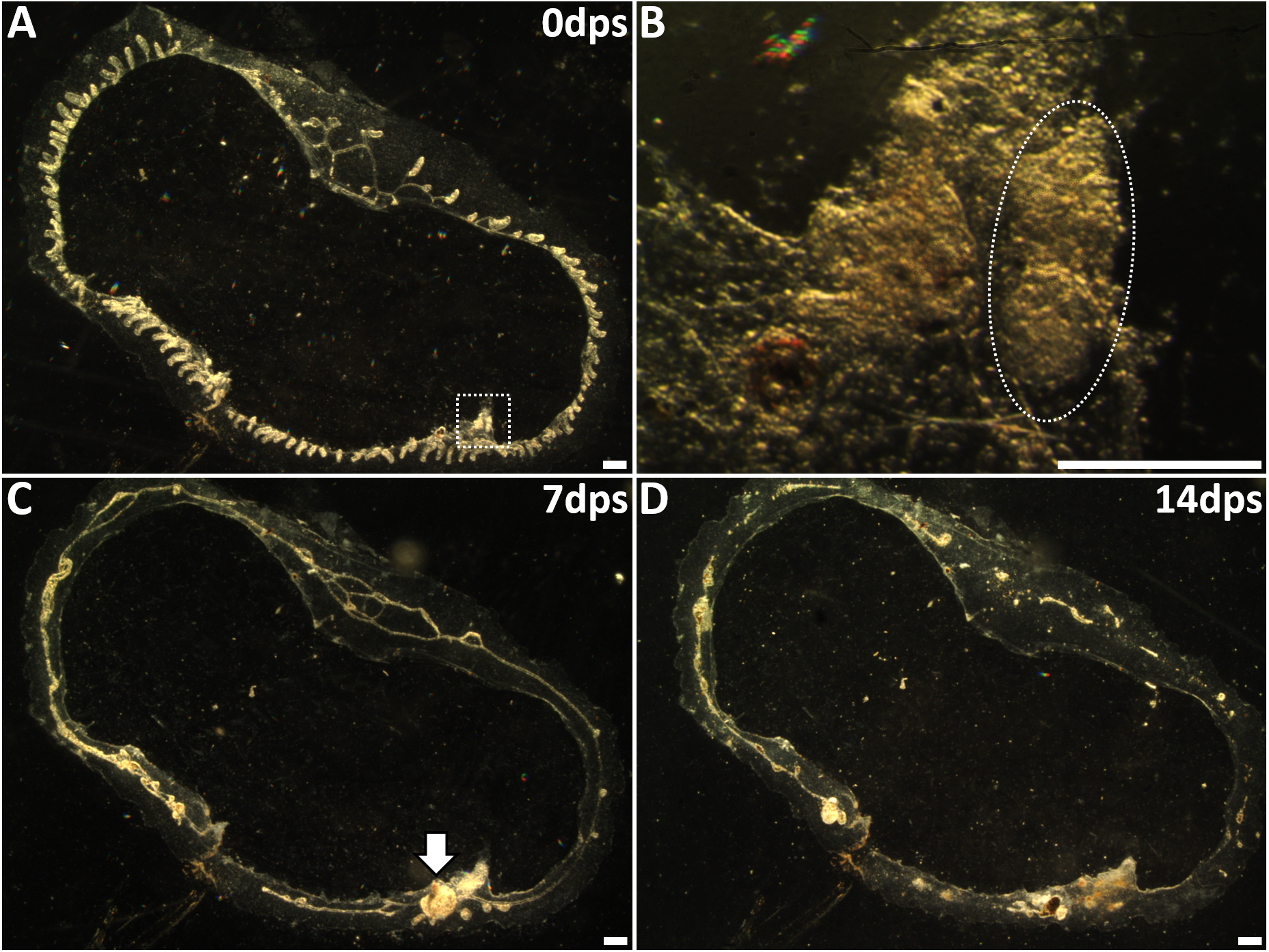

Supplement: Supplementary file 14 — Additional file 14: Figure S8. Assessing the potential for whole body regeneration from damaged secondary bud. A Post-surgery darkfield image of colony of B. schlosseri with a single secondary bud left behind that has been fragmented through the application of external pressure via forceps. B Magnified view of fragmented secondary bud from panel A. C Secondary bud developed a heartbeat at day 7. D Secondary bud resorbs by day 14, blood flow has ceased, and the vasculature has collapsed. dps = days post-surgery. Scale bars = 0.5 mm. [file 13227_2021_185_MOESM14_ESM.tif]

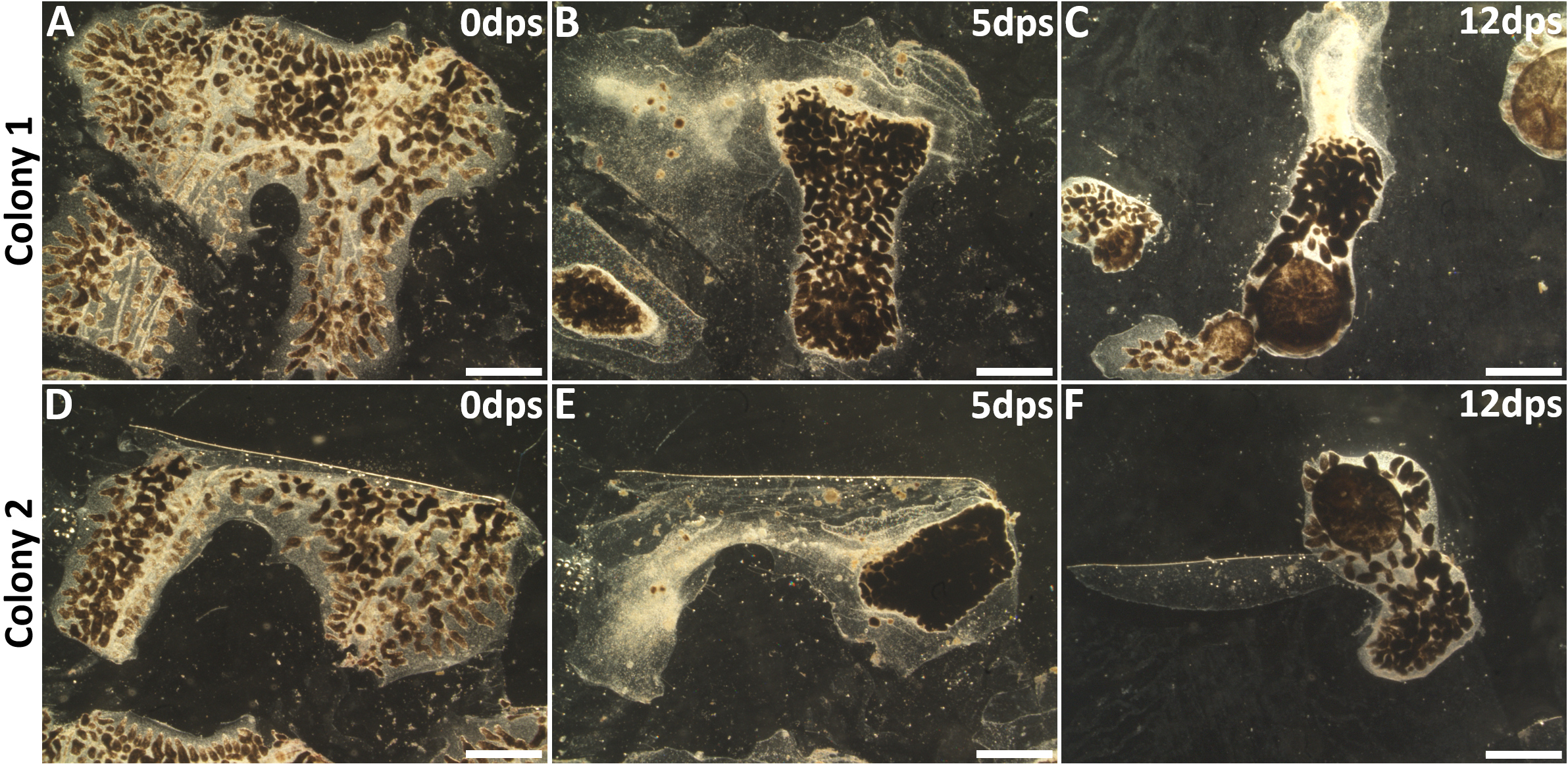

Supplement: Supplementary file 16 — Additional file 16: Figure S9. WBR from large vascular beds in Botrylloides diegensis. A–C A single zooid regenerating from a relatively large patch of vasculature. D–F Solitary zooid regeneration from two patches of ampullae connected by a single blood vessel. dps = days post-surgery. Scale bars = 2 mm. [file 13227_2021_185_MOESM16_ESM.tif]

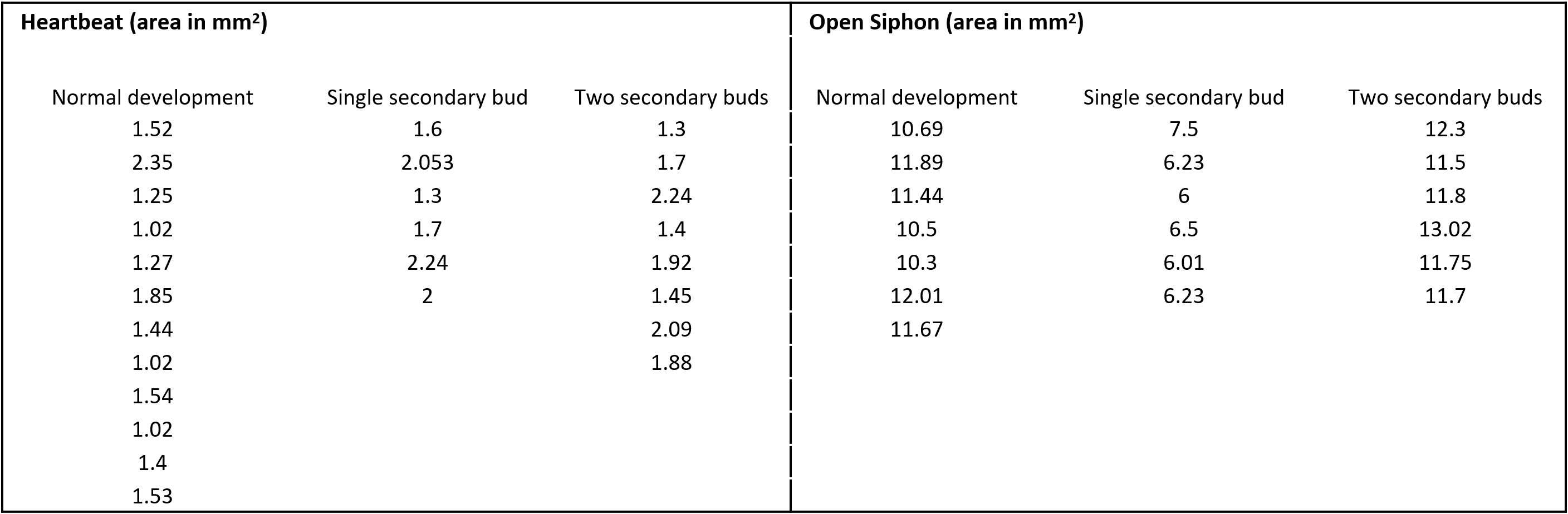

Supplement: Supplementary file 17 — Additional file 17: Table S3. Raw data for Fig. 8. Measured values used to create box plot shown in Fig. 8. Area values collected using ImageJ software [58]. [file 13227_2021_185_MOESM17_ESM.tif]

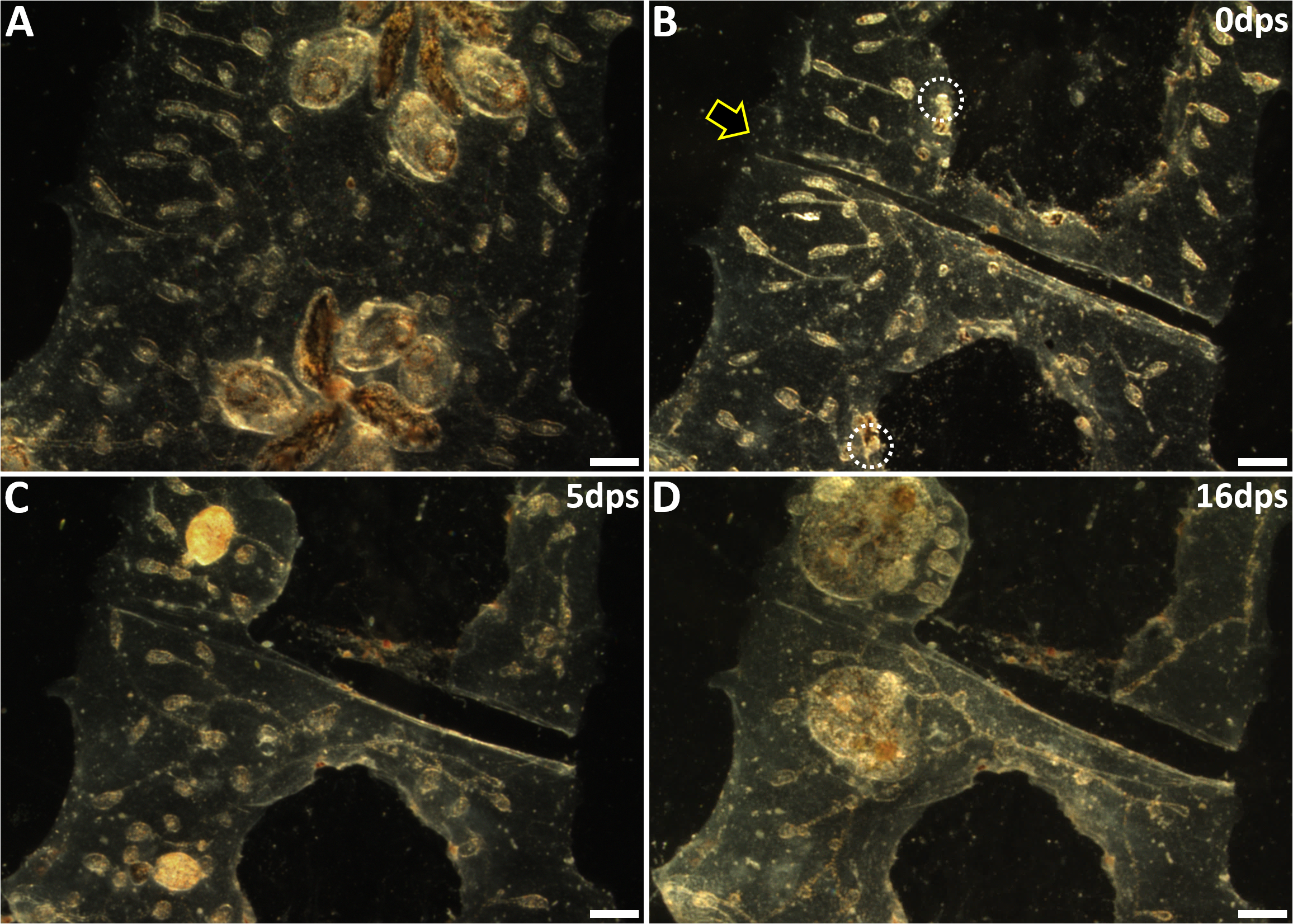

Supplement: Supplementary file 18 — Additional file 18: Figure S10. Post-surgery secondary buds develop independently while sharing tunic but not blood in Botryllus schlosseri. A Darkfield image of two systems within the same colony. B One secondary bud was isolated from each system (white circles). Two systems were left partially connected via the tunic (black arrow); however, the blood vasculature was removed from the region between them. C By day 5 each bud had developed a beating heart. D Both buds developed into filter-feeding adults. dps = days post-surgery. Scale bars = 0.5 mm. [file 13227_2021_185_MOESM18_ESM.tif]

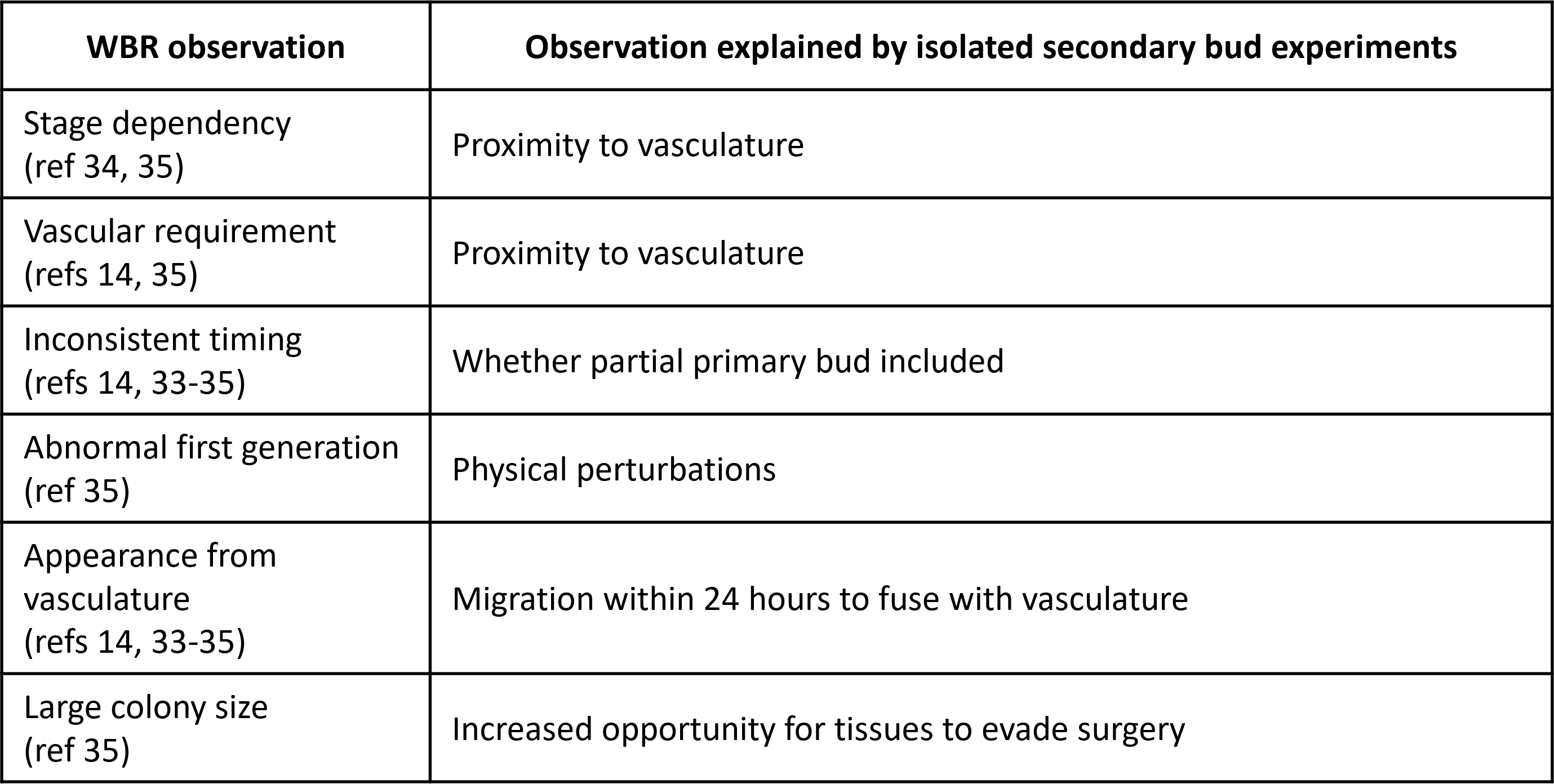

Supplement: Supplementary file 19 — Additional file 19: Table S4. Summary of how secondary bud isolation events explain WBR observations. The characteristics and requirements for WBR in Botryllus schlosseri match six observations when only a single secondary bud is isolated with vascular tissues after removal of all zooids and other buds. [file 13227_2021_185_MOESM19_ESM.tif]
